# Characterization of an HPV33 natural variant with enhanced transcriptional activity suggests a role for C/EBPβ in the regulation of the viral early promoter

**DOI:** 10.1038/s41598-019-41102-7

**Published:** 2019-03-25

**Authors:** Jennifer Alvarez, David Gagnon, François Coutlée, Jacques Archambault

**Affiliations:** 1 Department of Microbiology and Immunology, McGill University, Montreal, QC Canada; 2 Division of Experimental Medicine, McGill University, Montreal, QC Canada; 3 Institut de recherches cliniques de Montréal (IRCM), Montreal, QC Canada; 4 Department of Biochemistry and Molecular Medicine, Université de Montréal, Montreal, QC Canada; 5 Centre de Recherche et Département de Microbiologie Médicale et Infectiologie, Centre Hospitalier de l’Université de Montréal (CHUM), Université de Montréal, Montréal, QC Canada

## Abstract

The Long Control Region (LCR) of the human papillomavirus (HPV) genome encompasses the early promoter (EP) that drives expression of the viral oncogenes in infected cells and HPV-associated cancers. Here, we report on a natural variant of HPV33 that displays higher EP activity than the prototype in transfected C33A and HeLa cervical carcinoma cells, and in the osteosarcoma U2OS cell line which supports replication of HPV episomes. This increased promoter activity was ascribed to a single nucleotide variation in the LCR, T7791C, in a putative binding site for the transcription factor C/EBPβ. T7791C abrogated binding of recombinant C/EBPβ to this site *in vitro* and stimulated the EP *in vivo*, suggesting that it abrogates a negatively-acting regulatory element. A second C/EBPβ binding site was identified *in vitro* that activated the EP *in vivo* and whose function and location in the epithelial-specific enhancer is shown to be conserved in the highly prevalent HPV18. These results suggest that C/EBPβ is both an activator and a repressor of the HPV33 EP, acting via two distinct binding sites. Prediction of C/EBPβ sites in the LCR of 186 HPV types suggests that C/EBPβ regulation of the EP is common among high‐risk viruses from the α genus.

## Introduction

Persistent infections by high-risk human papillomaviruses (HR-HPVs) are associated with an increased risk of developing cervical cancer and other malignancies of the anogenital area, as well as a subset of head-and-neck cancers affecting the oropharynx, tonsils and/or base of the tongue^1,2^. HPV16 and HPV18 are the most prevalent oncogenic types, being responsible for 70–80% of all HPV-associated cancers. The remaining cases are caused by several types including HPV33, which accounts for 3–5% of all HPV-associated cancers worldwide. The two viral oncogenes, E6 and E7, not only promote tumorigenesis by antagonizing the p53 and pRb pathways, respectively, but remain essential for HPV-transformed cells to proliferate and survive, as first demonstrated in the HPV18-transformed HeLa cell line^3^. E6 and E7 are expressed from the HPV early promoter (EP) located within the regulatory part of the viral genome known as the long control region (LCR). The LCR contains binding sites for several cellular transcription factors such as Sp1 and AP-1 and for the viral E2 protein, a transcriptional repressor of the EP whose inactivation in HPV-associated cancers results in depression of the promoter and increased E6 and E7 expression^4,5^. Thus, apart from the repressive effect of E2 that is often lost in HPV-transformed cells, expression of E6 and E7 from the EP is entirely dictated by cellular transcription factors.

One of the factors that has been shown to regulate the HPV EP is C/EBPβ (CCAAT/Enhancer-binding Protein β), a ubiquitous member of the CCAAT family of transcription factors and an important regulator of genes involved in immunity, cell proliferation and differentiation (reviewed in^6^ and^7^). Of relevance to HPV, C/EBPβ is required for the differentiation of keratinocytes in stratified squamous epithelia^8^ and the activation of the viral late promoter in the most differentiated cell layers^8,9^. Three C/EBPβ isoforms have been identified: the liver-enriched activator proteins LAP* and LAP (herein termed LAP only) and the liver-enriched inhibitory protein LIP^7,10^. The latter lacks the transactivation domains but retains the ability to bind DNA via its basic leucine-zipper (bZIP) domain and to heterodimerize with LAP. The relative expression of LAP and LIP (LAP/LIP ratio) influences the ability of C/EBPβ to activate or repress cellular promoters^6,7^. Although C/EBPβ preferentially binds as a homodimer to the consensus sequence 5′-ATTGCGCAAT-3′^5,11^, it can also form heterodimers with other C/EBP family members and bZIP factors such as CREB, NF-κB, and ATF^7^ to bind composite DNA target sites and regulate an extended range of promoters.

Regulation of the EP by C/EBPβ has been examined primarily in HPV11, HPV16, and HPV18 (reviewed in^5^). Studies on HPV11 suggested that C/EBPβ represses the EP in PHK, either by binding to specific target sites in the LCR^12,15^ or independently of these sites when overexpressed by transfection^13^. Consistent with C/EBPβ acting as a repressor in PHK, a study on HPV31 showed that LIP is the predominant isoform in these cells and that its expression decreases upon keratinocyte differentiation such as to favor LAP-induced transactivation of the viral late promoter^8^. The HPV18 EP has been extensively studied in HeLa cells and shown to be activated by the assembly of a C/EBPβ-YY1 complex on the so-called “switch region” of the LCR^14,16^ but also repressed by overproduction of C/EBPβ independently of this region^17^. Repression of the EP by overexpressed C/EBPβ was also demonstrated for HPV16 in PHK, HeLa and HPV16-transformed CasKi cells^9,18^. In contrast and for reasons that remain elusive, C/EBPβ overexpression resulted in transactivation of the HPV16 and HPV11 EPs in the HPV-negative C33A cervical carcinoma cell line^15,19^. Thus, it appears that C/EBPβ regulates the EP through direct and indirect mechanisms and that its ability to function as a repressor or an activator of early gene transcription varies between cellular models.

Naturally-occurring polymorphisms in the LCR can alter the activity of the EP. Indeed, pioneering studies on HPV16^20,21^ and HPV18^22^ demonstrated that variations in the LCR can modulate the activity of the EP 2- to 3-fold in reporter-gene assays, but that these small differences in transcriptional activity are sufficient to alter the replication of viral episomes through changes in the expression of the replication proteins E1 and E2^20–22^. From a methodological standpoint, these studies also indicated that the EP activity measured from the LCR in reporter-gene assays is reflective of the activity of the promoter in the context of the full viral genome.

HPV33, the subject of this study, is one of the most prevalent types after HPV16 and -18 in Canada and in the world^23,24^. HPV33 accounts for 3–5% of all HPV-associated cancers of the female anogenital area. Several HPV33 isolates have been identified and classified in two main phylogenetic lineages termed A and B, with the former being subdivided in the two sublineages A1 and A2. The first HPV33 isolate, known as the prototype, belongs to the A1 sublineage. Previous epidemiological studies revealed that the prototype and other A1-sublineage isolates are associated with cervical intraepithelial neoplasia of grades 2–3 (CIN2/3)^25^ and cervical cancer^26^, while viruses from the A2-sublinage and B-lineage are less oncogenic but give rise to infections that persist longer by approximately 6-months^27^. These and our own epidemiological studies mentioned below prompted us to examine the transcriptional activity of HPV33 LCR variants to assess if the strength of the EP is associated with increased oncogenicity and/or lower persistence, and to identify specific variations that modulate transcription of the early genes. We recently reported that a 79 bp sequence that is present in all HPV33 isolates and is uniquely duplicated in the LCR of the prototype and A1-sublineage variants increases the activity of the EP approximately two-fold in PHK^28^. This finding suggested that the higher EP activity of A1-sublineage viruses, caused by the 79 bp duplication, contributes to their higher oncogenicity. Interestingly, our own epidemiological studies also revealed that the naturally occurring 79 del variation, which removes one of the two 79 bp regions and decreases EP activity in A2- and B-variants^28^, is associated with persistent infection^29^. Together, these results raised the possibility that HPV33 variants with higher EP activity may be more oncogenic while those with lower transcriptional activity may persist longer^28^. In further support of this notion, we observed that the C7732G variation that is present in some A2-sublineage variants and which we previously found to be associated with high grade squamous intraepithelial lesion (HSIL)^30^, also increases the activity of the HPV33 EP approximately 2-fold in PHK^28^.

In the course of our clinical investigations, we identified a rare B-lineage variant LCR, termed LCR10, in one out of 37 HPV33-infected individuals^29^. In this manuscript, we present our functional characterization of LCR10 showing that it displays higher EP activity than other B-lineage variants in undifferentiated PHK, the natural target cells of HPVs, as well as in HeLa cells as a model of an HPV-associated cancer (an HPV33 transformed cell line does not currently exist). LCR10 was also more active than other B-lineage LCRs in the C33A cell line that has been used to study the EP from other HPV types, and in the U2OS osteosarcoma cell line which was shown in recent years to support the replication and maintenance of HPV episomes from several types, including HPV33, and the synthesis of early mRNAs from the EP similar to those found in PHK^31–34^. Our results indicate that the higher EP activity of LCR10 in transformed cell lines is due to a single nucleotide variation, T7791C, which abrogates a binding site for C/EBPβ that represses the EP. A second C/EBPβ site was also identified that activates the EP and whose function and location in the LCR are conserved in HPV18. These results indicate that the HPV33 EP is regulated negatively and positively by C/EBPβ via two distinct regulatory elements, and that T7791C results in derepression of the promoter. Lastly, we also provide bioinformatic evidence that regulation of the EP by C/EBPβ is a common feature of high-risk HPV types from the α genus.

## Results

### Identification of LCR10, a B-lineage variant LCR with enhanced transcriptional activity

**Figure 1:**
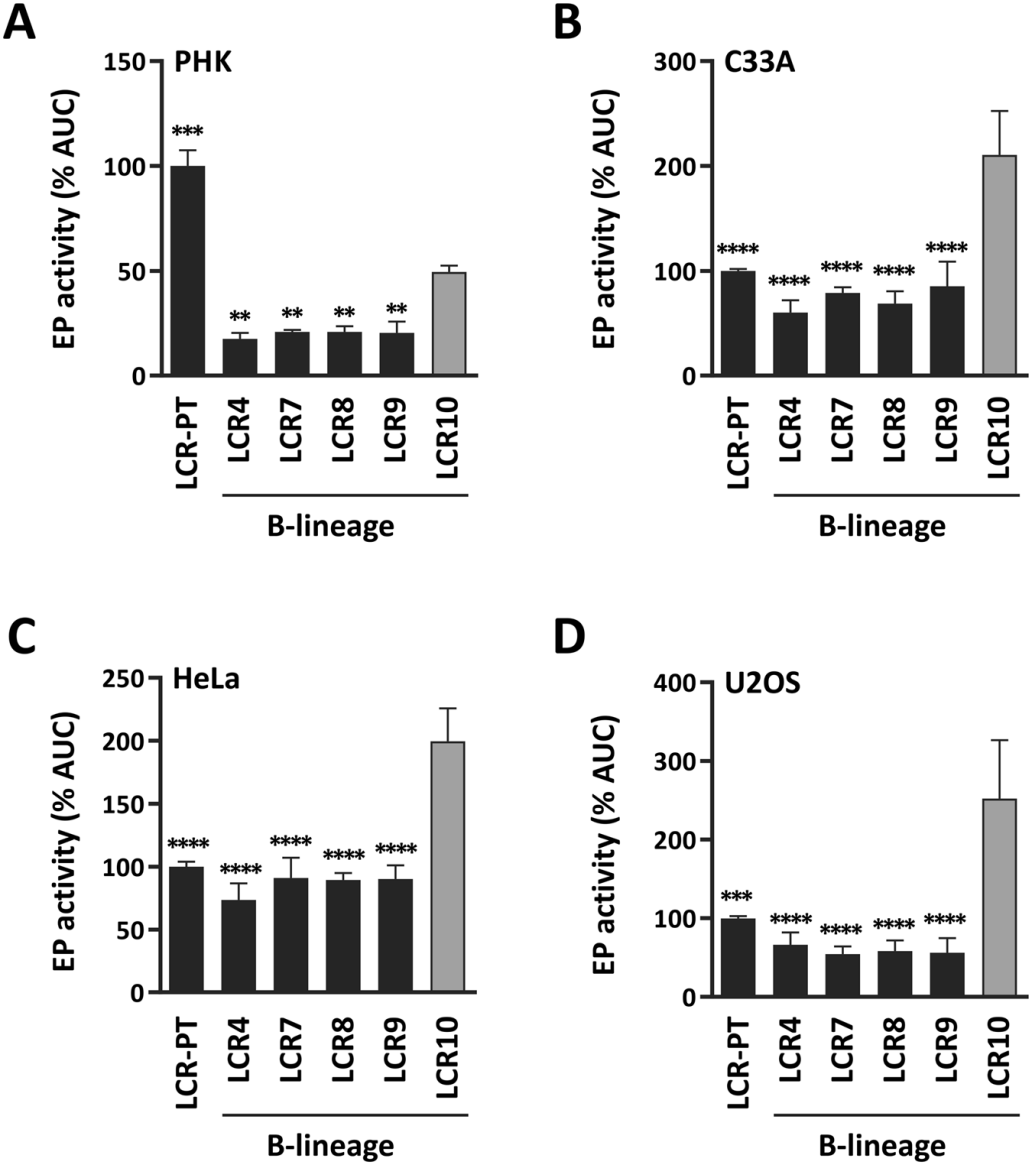
Transcriptional activity of LCR10 in PHK and transformed cell lines. (**A**–**D**) Bar graphs showing the transcriptional activities of LCR10 compared to those of the prototype (LCR-PT) and of other B-lineage variant LCRs (LCR4, LCR7, LCR8 and LCR9), measured by luciferase reporter-gene assays in PHK (**A**), C33A (**B**), HeLa (**C**) and U2OS (**D**) cells. Each bar represents the activity determined from a minimum of 3 independent dose-response curves performed in duplicates and reported as the average AUC value, as described in the Methods section. The activity of LCR-PT was used as the reference and set to 100%. Standard deviations are indicated by error bars. Transcriptional activities that differ from that of LCR10 were identified using a one-way ANOVA followed by Dunnett’s post-hoc analysis and are indicated according to their significance: *p ≤ 0.05, **p ≤ 0.01, ***p ≤ 0.001, ****p ≤ 0.0001.

We previously characterized the LCR of several naturally-occurring HPV33 variants from the A and B linages and identified specific nucleotide changes that either enhanced or decreased the transcriptional activity of the EP in luciferase-reporter gene assays^28^. The LCRs of the four B-lineage variants examined in this previous study, termed LCR4, LCR7, LCR8 and LCR9, contained 17 or 18 variations compared to the prototype LCR (LCR-PT) when considering substitutions, insertions and deletions as individual variations (Table 1). LCR4, LCR7, LCR8 and LCR9 showed comparable EP activities relative to each other in PHK and HeLa cells^28^, and this observation is extended to C33A and U2OS cells in this study (Fig. 1). Although this indicated that the strength of the EP is comparable among B-lineage variants, this conclusion is not absolute as we recently identified a fifth LCR from the B lineage, LCR10, with significantly higher transcriptional activity (Fig. 1A–D). LCR10 was even more active than the prototype (LCR-PT) in the three cancer cell lines C33A, HeLa and U2OS, but not in PHK. Sequencing of LCR10 revealed that it contains 19 nucleotide variations compared to the prototype (Table 1). Three of those variations, T7365C, C7531T and T7791C are not found in other B-lineage LCRs and may therefore account for the higher transcriptional activity of LCR10, as tested below.

**Table 1:** Nucleotide variations in B-lineage LCRs.

Position	Variations																					
	7116	7128	7174	7182	7198	7227	7362	7365*	7404	7412	7422	7424	7425	7442	7454	7481	7529	7531*	7535	7595	7791*	18*
LCR-PT	T	T	T	G	T	G	T	T	T	TA	G	A	C	G	G	T	—	C	G	79 bp	T	G
LCR4	G	C	C	C	C	A	C	—	del	—	T	G	T	A	A	G	—	—	A	del	—	A
LCR7	G	C	C	C	C	A	C	—	A	—	T	G	T	A	A	G	—	—	A	del	—	A
LCR8	G	C	C	C	C	A	C	—	A	—	T	G	T	A	A	G	Ins	—	A	del	—	A
LCR9	G	C	C	C	C	A	C	—	A	del	T	G	T	A	A	G	—	—	A	del	—	A
LCR10	G	C	C	C	C	A	C	C	A	—	T	G	T	T	A	G	—	T	A	del	C	—

Variant positions of the LCR are numbered according to the sequence of the HPV33 prototype (PT) genome (GenBank M12732.1). “Ins” refers to the insertion of the sequence CCCTAATA at position 7529 of LCR8. Deletions are abbreviated “del”. Positions identical to the prototype are indicated by hyphens. Positions that differ between LCR7 and LCR10 are indicated by asterisks.

The data presented Fig. 1 also shows that the transcriptional activities of the B-lineage LCRs are much lower in PHK than in the transformed cell lines, when compared to LCR-PT. This is because all B-lineage LCRs, including LCR10, contain the 79 del variation that removes one copy of the duplicated 79 bp region present in the prototype (Table 1). We previously reported that 79 del reduces the activity of the EP approximately 2-fold in PHK but has little to no effect in HeLa cells^28^. We also determined in this study that the EP is not significantly affected by 79 del in C33A and U2OS cells (Supplementary Fig. S1). Thus, the presence of 79 del in the B-lineage LCRs contributes to their low transcriptional activity in PHK, relative to the prototype.

### T7791C accounts for the enhanced EP activity of LCR10 in C33A, HeLa and U2OS cells

**Figure 2:**
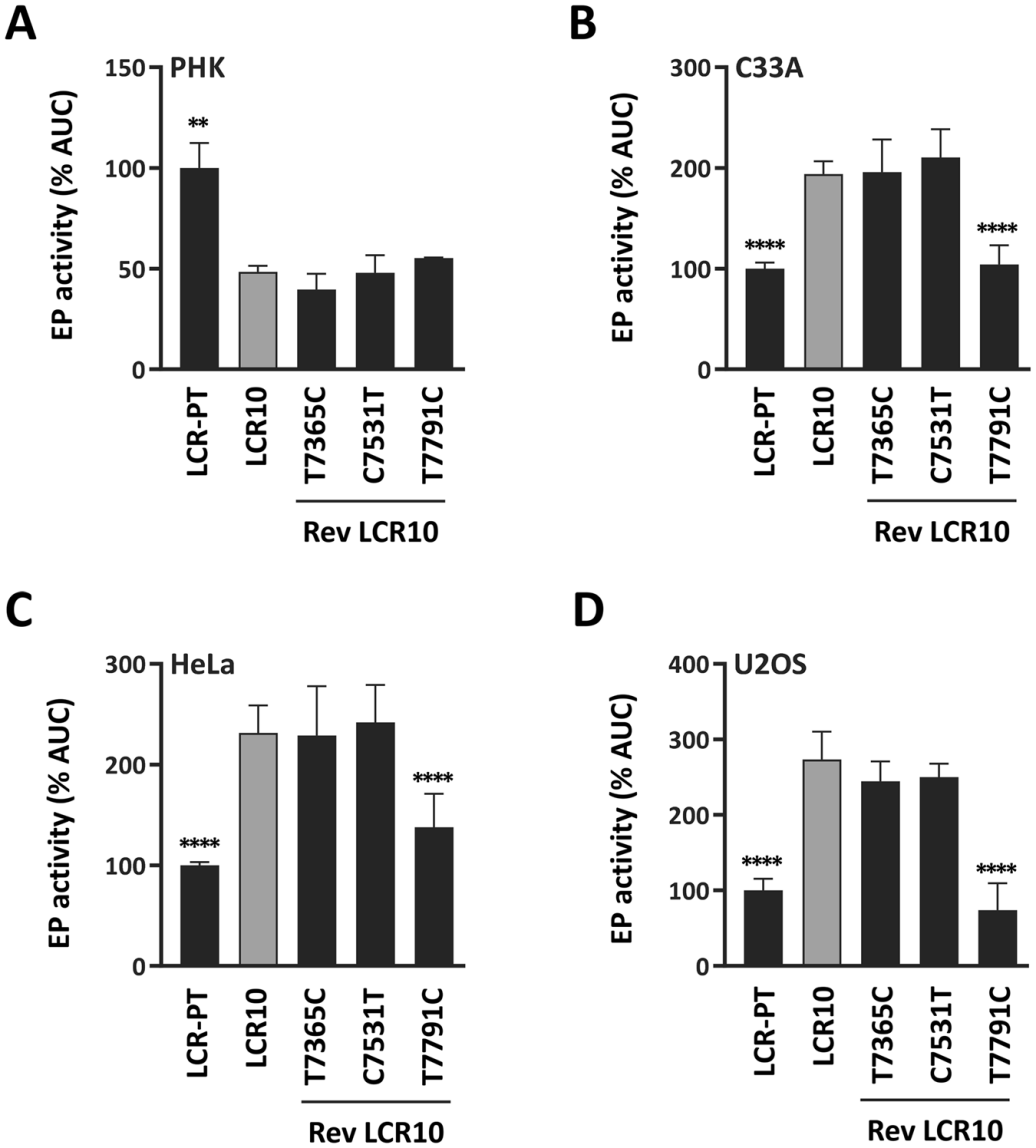
T7791C accounts for the higher activity of LCR10 in C33A, HeLa and U2OS cells. (**A**–**D**) Bar graphs showing the transcriptional activities of LCR-PT, LCR10, and of LCR10 revertants (Rev LCR10) in which either T7365C, C7531T or T7791C has been reverted, as indicated. Transcriptional activities were measured by luciferase reporter-gene assays in PHK (**A**), C33A (**B**), HeLa (**C**) and U2OS (**D**) cells and reported as described in the legend of Fig. 1 relative to the activity of LCR-PT (100%). Transcriptional activities that differ significantly from that of LCR10 are indicated.

The results presented above suggested that the T7365C, C7531T and/or T7791C variations may contribute to the higher transcriptional activity of LCR10 compared to the other four B-lineage LCRs. To determine the contribution of these three variations to the activity of LCR10, they were reverted individually back to the sequence of the prototype, and the transcriptional activities of the resulting revertant LCRs measured in reporter-gene assays. As shown in Fig. 2A, reversion of each variation did not significantly change the activity of LCR10 in PHK. In C33A, HeLa and U2OS cells, however, reversion of T7791C decreased the activity of LCR10 by 40–60%, down to the levels measured for the prototype (Fig. 2B–D). Reversion of T7365C and C7531T, in contrast, had little to no effect (Fig. 2B–D). Collectively, these results indicate that T7791C is the main variation that activates the LCR10 EP in C33A, HeLa and U2OS cells.

### The combination of T7791C, T7365C and C7531T in LCR10, and the G18A variation in other B-lineage LCRs repress the EP in PHK

**Figure 3:**
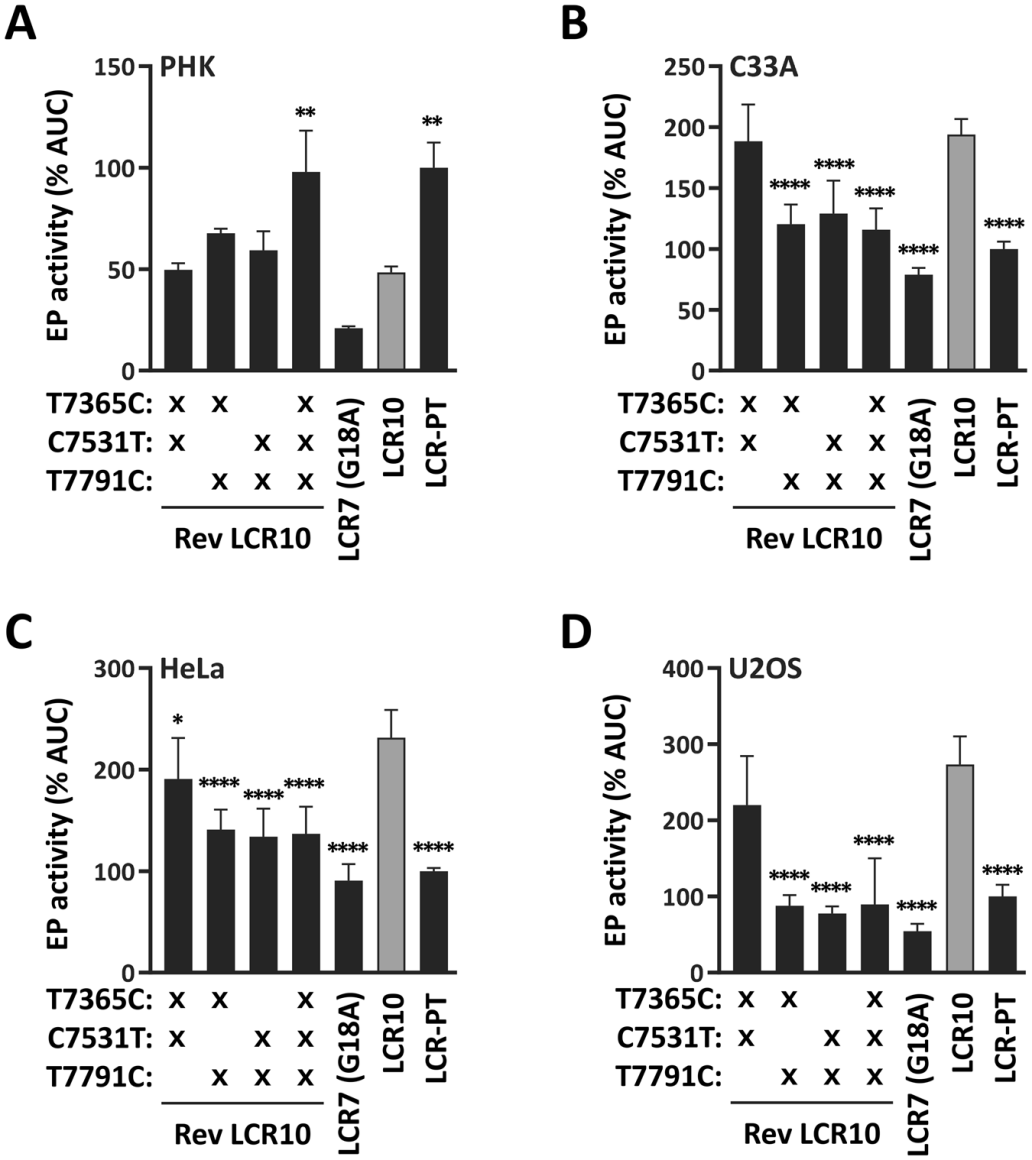
Repression of the EP in PHK either by the combination of T7365C, C7531T and T7791C in LCR10 or by G18A in the LCR of other B-lineage variants. (**A**–**D**) Bar graphs showing the transcriptional activities of LCR-PT, LCR7, LCR10, and of LCR10 revertant (Rev LCR10) in which T7365C, C7531T or T7791C were reverted in double or triple combinations. Variation(s) that were reverted in each LCR10 revertant are indicated by an x. LCR7 differs from the triple LCR10 revertant only by the presence of G18A. Transcriptional activities were measured by luciferase reporter-gene assays in PHK (**A**), C33A (**B**), HeLa (**C**) and U2OS (**D**) cells and reported as described in the legend of Fig. 1 relative to the activity of LCR-PT (100%). Transcriptional activities that differ significantly from that of LCR10 are indicated.

The fact that individual reversion of T7791C, T7365C or C7531T did not alter the activity of LCR10 in PHK prompted us to investigate if reverting these variations in combination would have an effect. While none of the double revertant LCRs differed from LCR10, the triple revertant showed significantly increased activity (Fig. 3A). These results suggest that T7791C, T7365C and C7531T act together to repress the activity of the LCR10 EP in PHK. In C33A, HeLa and U2OS cells, only the revertant LCRs lacking T7791C showed lower activity, thus confirming the results from Fig. 2 that T7791C activates the LCR10 EP in these cells.

The LCR10 triple revertant (i.e. lacking T7365C, C7531T and T7791C) is nearly identical in sequence to LCR7, the only difference being the presence of an additional variation in LCR7, G18A. This allowed the effect of G18A on the EP to be examined by comparing the transcriptional activity of LCR7 to that of the LCR10 triple revertant. The transcriptional activity of LCR7 was approximately 5-fold weaker than that of the LCR10 triple revertant in PHK (p ≤ 0.01), indicating that G18A represses the EP of LCR7 in these cells. G18A also decreased, albeit slightly, the EP activity of LCR7 in C33A (p ≤ 0.001) and HeLa (p ≤ 0.01) cells.

In summary, these results indicated that T7791C represses the EP in combination with T7365C and C7531T in PHK, and is sufficient to activate the EP in C33A, HeLa and U2OS cells. They also showed that the absence of G18A in LCR10 contributes to its higher transcriptional activity compared to the other B-lineage variants which all contain this variation (Table 1).

### The T7791C variation affects one of two binding sites for the transcription factor C/EBPβ

**Figure 4:**
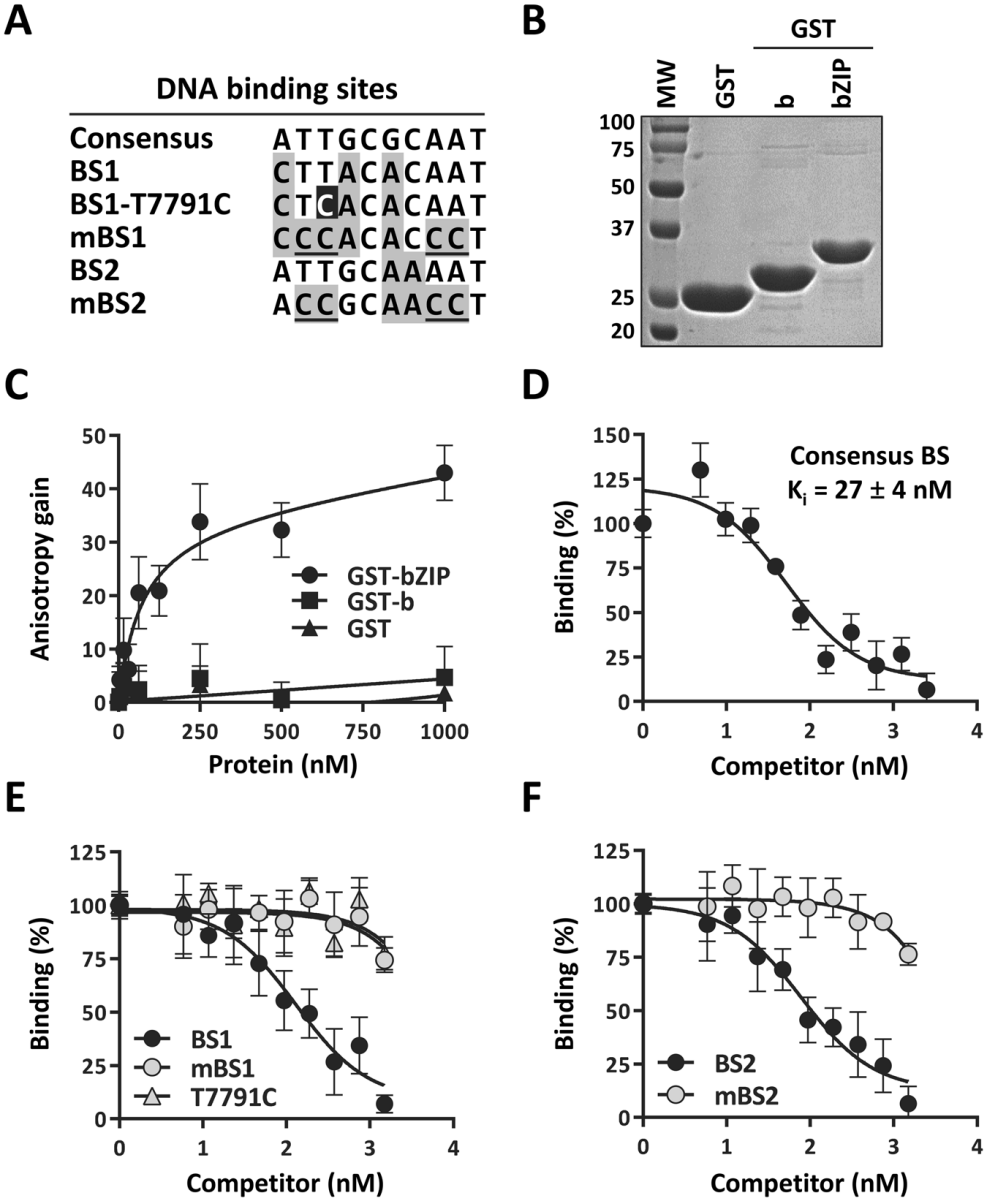
DNA-binding activity of C/EBPβ measured by fluorescence anisotropy *in vitro*. (**A**) Sequences of the consensus C/EBPβ binding site and of the wild type, variant and mutant BS1 and BS2 sites found in the HPV33 LCR. Nucleotides mutated in mBS1 and mBS2 are underlined and those differing from the consensus sequence shaded in grey. The T7791C variation is highlighted in black. (**B**) 12% SDS-PAGE analysis of the purified GST-fusion proteins spanning the C/EBPβ basic (GST-b) and basic plus leucine-zipper regions (GST-bZIP). Three μg of each protein and of GST alone were analyzed and stained with Coomassie. The size of molecular weight standards (MW, in kDa) are shown on the left of the gel. (**C**) Binding isotherms showing the concentration-dependent gain in anisotropy resulting from the interaction of GST-bZIP with a fluorescein-labeled DNA probe containing BS1. GST-b and GST were used as negative controls. Binding reactions were performed with increasing concentrations of each protein, from 3.9 to 1000 nM in 2-fold increments, and 15 nM of probe. Each data point is the average of duplicates values measured in 3 independent experiments (n = 6). Error bars represent standard deviations. (**D**) Inhibition curve showing the ability of an unlabeled consensus C/EBPβ site (at concentrations from 5.9 to 1500 nM in 2-fold increments) to compete the binding of GST-bZIP (125 nM) to the BS1 probe (15 nM). The K_i_ value calculated from this curve is indicated. Changes in anisotropy are reported as a percent of the control value (% binding) measured in absence of competitor DNA. Each data point is the average of duplicate values obtained from at least 3 independent experiments (n = 6). Error bars represent standard deviations. (**E**,**F**) Inhibition curves obtained with unlabeled oligonucleotides containing BS1, the variant T7791C site, or the mutant mBS1 site (**E**) or containing the wild type or mutant BS2 (**F**). These competition experiments were performed as described in panel D.

T7791C changes a DNA sequence that resembles a binding site for the transcription factor C/EBPβ; we termed this site BS1 in LCR-PT and BS1-T7791C in LCR10 (sequence shown in Fig. 4A). To test if C/EBPβ can interact with this site, a biochemical DNA binding assay based on fluorescence anisotropy was developed. C/EBPβ contains a basic region (b) and a leucine zipper domain (ZIP) that contacts DNA and mediates its dimerization, respectively, and which are both needed for specific and high-affinity binding to DNA^7^. A C/EBPβ fragment encompassing the b and ZIP regions (bZIP) was bacterially expressed as a fusion to glutathione-S-transferase in bacteria and purified (GST-C/EBPβ-bZIP, Fig. 4B). A shorter fragment containing only the b region was similarly produced as a negative control (GST-C/EBPβ-b, Fig. 4B). The ability of these recombinant proteins to bind a fluorescein-labeled DNA probe containing BS1 was then determined. Titration of GST-C/EBPβ-bZIP resulted in a dose-dependent increase in anisotropy indicative of its binding to the BS1 probe (Fig. 4C). From this binding isotherm, a dissociation constant (K_D_) of 62 ± 3 nM was measured for the interaction of GST-C/EBPβ-bZIP with BS1. This interaction was specific as neither GST-C/EBPβ-b nor GST alone could bind to the probe (Fig. 4C).

As further validation of the assay, we verified that an unlabeled oligonucleotide containing a single C/EBPβ consensus site (5′-ATTGCGCAAT-3′)^35^ could inhibit the binding of GST-C/EBPβ-bZIP to the BS1 probe in a dose-dependent manner and measured a K_i_ value of 27 ± 4 nM (Fig. 4D and Table 2). Mutagenesis of this palindromic consensus site indicated that GST-C/EBPβ-bZIP binds with higher affinity to sequences in which one of the two inverted repeats contains T residues at positions 2 and 3, and C at position 5 (5′-ATTGC-3′; critical positions underlined) (Supplementary Fig. S2).

**Table 2:** Affinities (K_i_) of C/EBPβ homodimers for different sites.

Competitor DNA	Sequence* (5′-3′)	K_i_ + SD (nM)
Consensus	ATTGCGCAAT	27 ± 4
BS1	C..A.A....	83 ± 29
BS1-T7791C	C.GA.A....	>1000
mBS1	CCCA.A.CC.	>1000
BS2	.....AA...	66 ± 19
mBS2	.CC.TA.CC.	>1000
HPV18 BS2L	C....AT..C	154 ± 37

*Dots indicate positions identical to the consensus.

Next, we measured the affinities (K_i_) of GST-C/EBPβ-bZIP for competitor oligonucleotides containing either the binding site found in LCR-PT (BS1), the variant BS1 (BS1-T7791C), or a defective BS1 that was inactivated by four mutations (mBS1; negative control) (Fig. 4A). GST-C/EBPβ-bZIP bound to BS1 with an affinity of 83 ± 29 nM (Fig. 4E and Table 2), which is only 3-fold lower than its affinity for the consensus site. Importantly, C/EBPβ-bZIP bound very poorly to the T7791C variant site and to the negative control mBS1 site (K_i_ > 1000 nM, Fig. 4E and Table 2). These results showed that C/EBPβ can bind specifically to BS1 *in vitro* but that this site is inactivated by the T7791C variation and mBS1 mutation.

To determine if the HPV33 LCR contains other C/EBPβ binding sites, in addition to BS1, we scanned its nucleotide sequence using a C/EBPβ position-weight matrices (PWMs) from the JASPAR database^36^ that was derived from genome-wide chromatin-immunoprecipitation (ChIP) studies performed by the ENCODE consortium (https://www.encodeproject.org/ [2018]). This PWM identified BS1, as expected, and predicted another site which was termed BS2. Competition experiments confirmed the binding of GST-C/EBPβ-bZIP to BS2 with a K_i_ of 66 ± 19 nM, approximately 2.5-fold lower than for the consensus sequence (Fig. 4F and Table 2). This interaction was specific as it was abolished by a quadruple mutation in BS2 (mBS2; Fig. 4F and Table 2). Collectively, the *in vitro* studies presented above identified two high-affinity C/EBPβ binding sites in the HPV33 LCR, BS1 and BS2, and revealed that the former is inactivated by the T7791C variation in LCR10.

### Modulation of the HPV33 EP by C/EBPβ BS1 and BS2 in PHK and transformed cell lines

**Figure 5:**
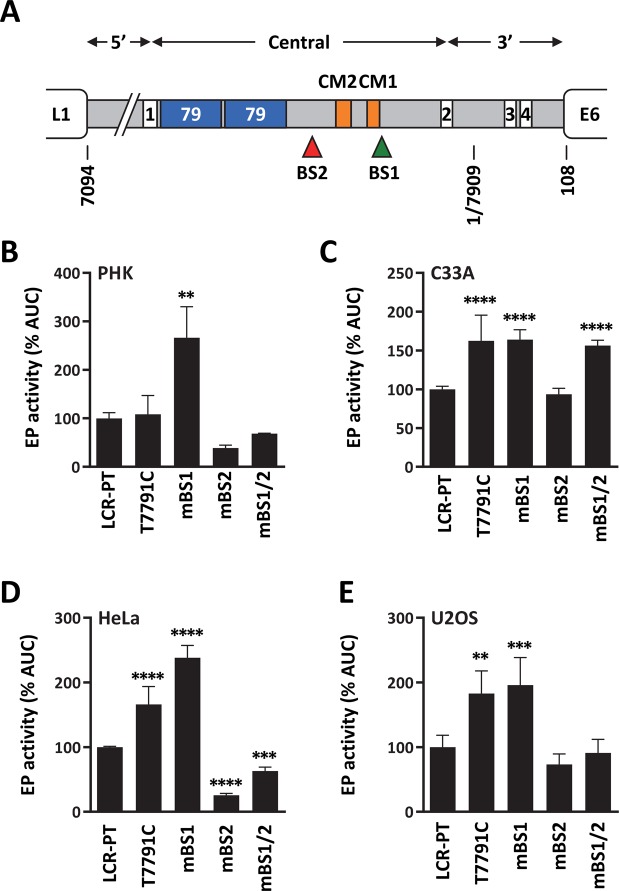
Effect of T7791C and of mutation of C/EBPβ BS1 and BS2 on the transcriptional activity of the prototype EP. (**A**) Schematic representation of the HPV33 prototype LCR (grey box) located between the L1 and E6 ORFs. The 4 E2 binding sites are indicated by white boxes numbered from 1 to 4, and the duplicated 79 bp region (79) by blue boxes. The C/EBPβ binding sites BS1 and BS2 are indicated by a green and a red triangle, respectively. Two conserved motifs in the LCR, which are termed CM1 and CM2 and are presented later in the manuscript, are indicated by orange boxes. The 5′, central and 3′ regions of the LCR are delineated by double headed arrows. (**B**–**E**) Bar graphs showing the transcriptional activities of LCR-PT and mutant derivatives carrying T7791C or the mBS1 and mBS2 mutations either alone or in combination, as indicated. Transcriptional activities were measured by luciferase reporter-gene assays in PHK (**B**), C33A (**C**), HeLa (**D**) and U2OS (**E**) cells and reported as described in the legend of Fig. 1 relative to the activity of LCR-PT (100%). Transcriptional activities that differ significantly from that of LCR-PT are indicated.

The locations of BS1 and BS2 in the HPV33 LCR is shown in Fig. 5A, relative to the positions of the four E2-binding sites. To test the contribution of these C/EBPβ sites to the activity of the HPV33 EP, they were inactivated in LCR-PT by the same mBS1 and mBS2 mutations described above (Fig. 4A), either individually or in combination. T7791C was also introduced alone into LCR-PT for comparison with mBS1. The results from luciferase reporter-gene assays presented in Fig. 5B–E show that mutation of BS1 (mBS1) increased the activity of LCR-PT in PHK (2.7-fold) and in the three cancer cell lines C33A (1.6-fold), HeLa (2.4-fold) and U2OS (2-fold). These results suggest that BS1 represses the EP in all four cell types. Like mBS1, T7791C also stimulated the EP in C33A, HeLa and U2OS cells. Surprisingly, however, T7791 had no effect in PHK. While clearly highlighting a functional difference between T7791C and mBS1 in PHK, this result is nevertheless consistent with the finding that reversion of T7791C in LCR10 had little effect on its transcriptional activity unless combined with reversion of T7365C and T7791 (Fig. 3A).

Mutation of BS2 (mBS2) showed a substantial effect in PHK and HeLa cells, where it decreased the activity of LCR-PT 2.6-fold and 4-fold, respectively. These results suggest that BS2 activates the EP in HeLa cells, and perhaps also in PHK although this result did not reach statistical significance. The negative effect of mBS2 in PHK became significant, however, when the combination mBS1 and mBS2 is compared to mBS1 alone; the double mutant LCR being nearly 4-fold less active than the mBS1 LCR (p ≤ 0.01). A similar observation was made in U2OS cells where the reduction in LCR-PT activity caused by mBS2 became significant only when analyzed in the context of mBS1 (compare mBS1 LCR vs mBS1/mBS2 LCR in Fig. 5E; p ≤ 0.001). In contrast, mBS2 had little to no effect in C33A cells even in combination with mBS1 (Fig. 5C). Although we did not investigate the mechanism underlying the inactivity of BS2 in C33A cells, we note that these cells are known to express little to no BRM and BRG1^37,38^, two ATPases of the SNF/SWI co-activator complex which interacts with the LAP* isoform of C/EBPβ and mediates its transactivation^39–41^. Thus, C33A cells may be partly defective in supporting C/EBPβ-mediated transactivation of viral and cellular promoters. In summary, the results presented above indicate that the transcriptional activity of the HPV33 EP is repressed by BS1 in all four cell types and activated by BS2 in PHK, HeLa and U2OS cells. These findings suggest that C/EBPβ is both an activator and a repressor of the HPV33 EP, acting via two separate regulatory elements.

### Modulation of the HPV33 EP by overexpression of the C/EBPβ LAP and LIP isoforms

**Figure 6:**
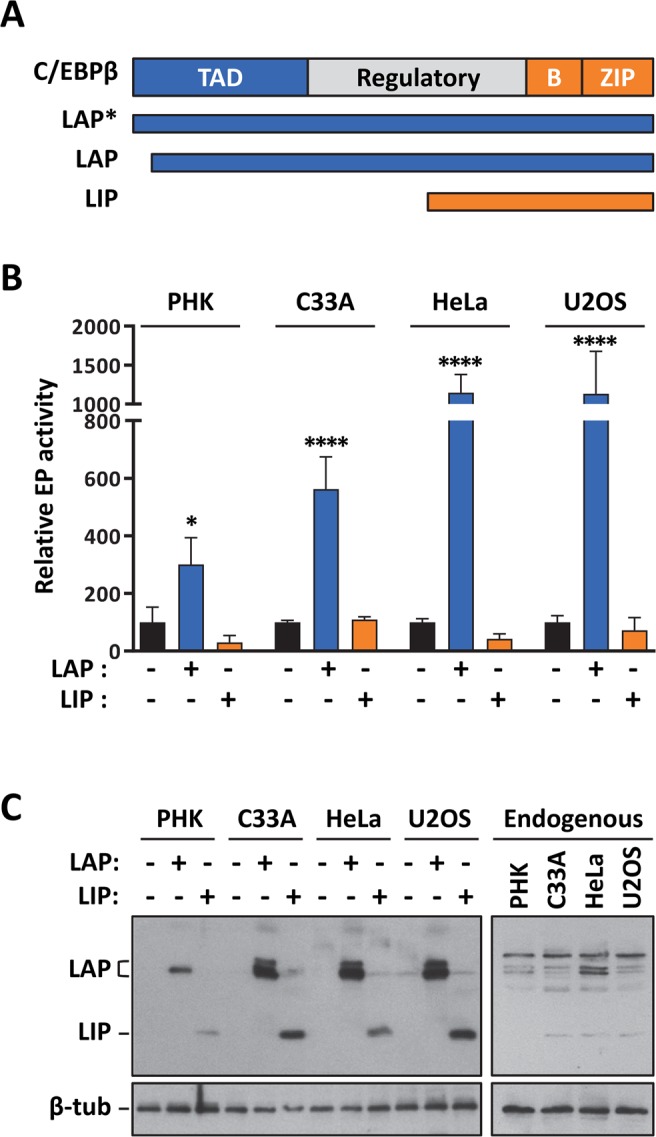
Expression levels of the C/EBPβ LAP and LIP isoforms in PHK, C33A, HeLa and U2OS cells and effect of their overproduction on the activity of the EP. (**A**) Schematic representation of C/EBPβ showing the location of the transactivation domain (TAD, in black), regulatory region (white) and of the DNA-binding and dimerization domain (grey) comprised of the basic (**B**) and leucine-zipper (Z) regions. The C/EBPβ fragments corresponding to the LAP, LAP* and LIP isoforms are indicated by thick black lines. (**B**) Western blots showing the expression levels of endogenous C/EBPβ LAP and LIP in PHK, C33A, HeLa and U2OS cells (left panel) and of overexpressed (exogenous) LAP and LIP in transfected HeLa cells (right panel). Mock transfected HeLa cells were used as a negative control. Both isoforms were detected using a rabbit polyclonal antibody against C/EBPβ. The bands corresponding to LAP and LIP are indicated on the right. Β-tubulin (β-tub) was used as a loading control. (**C**) Transcriptional activity of LCR-PT measured by reporter gene assays in PHK, C33A, HeLa and U2OS cells co-transfected (+) or not (−) with a plasmid expressing either LAP (5 ng) or LIP (10 ng), as indicated. In each cell type, bars represent the mean values of Rluc/Fluc ratios obtained from at least 3 independent experiments each performed in duplicates, as described in the Methods section, and normalized to the Rluc/Fluc value measured for LCR-PT in the absence of exogenous LAP or LIP, which was set to 100% as the reference. Values that differed from this reference were identified using a one-way ANOVA followed by Dunnett’s post-hoc analysis and are labeled according to their significance: *p ≤ 0.05, **p ≤ 0.01, ***p ≤ 0.001 and ****p ≤ 0.0001.

As mentioned in the Introduction, C/EBPβ has been shown to modulate the EP of HPV11, 18 and 16 when overexpressed. To test if this is also true for HPV33, we investigated the effect of overproducing the C/EBPβ LAP and LIP isoforms (Fig. 6A) on the activity of LCR-PT in reporter-gene assays. Ectopic expression of LAP increased the activity of the prototype EP in all four cell types (Fig. 6B), from approximately 3-fold in PHK to greater than 10-fold in HeLa and U2OS cells. LIP, in contrast, did not affect the EP in a statistically-significant manner although it appeared to repress it in PHK and HeLa cells, and perhaps also in U2OS cells (Fig. 6B). The overexpression of LIP and LAP in the four cell types was confirmed by Western blotting (Fig. 6C; left panel). Together, these results provided further evidence that C/EBPβ regulates the HPV33 EP by C/EBPβ. We emphasize, however, that these effects of exogenous LAP and LIP are likely the sum of direct effects on the LCR and of indirect ones on other transcription factors that modulate the EP.

The results presented so far revealed differences in how C/EBPβ regulates the HPV33 EP in the four cellular models. This led us to investigate the amount of endogenous C/EBPβ isoforms expressed in each cell type. The Western blot shown in Fig. 6C (right panel) revealed that PHK express very little LIP while HeLa cells have higher levels of LAP than the other cell types. Thus, the higher LAP/LIP ratio in PHK and HeLa cells may explain, at least in part, why mutation of the stimulatory BS2 site showed its largest effect in PHK and HeLa cells (Fig. 5).

### Prediction of C/EBPβ binding sites in the LCRs of 186 HPV types

**Figure 7:**
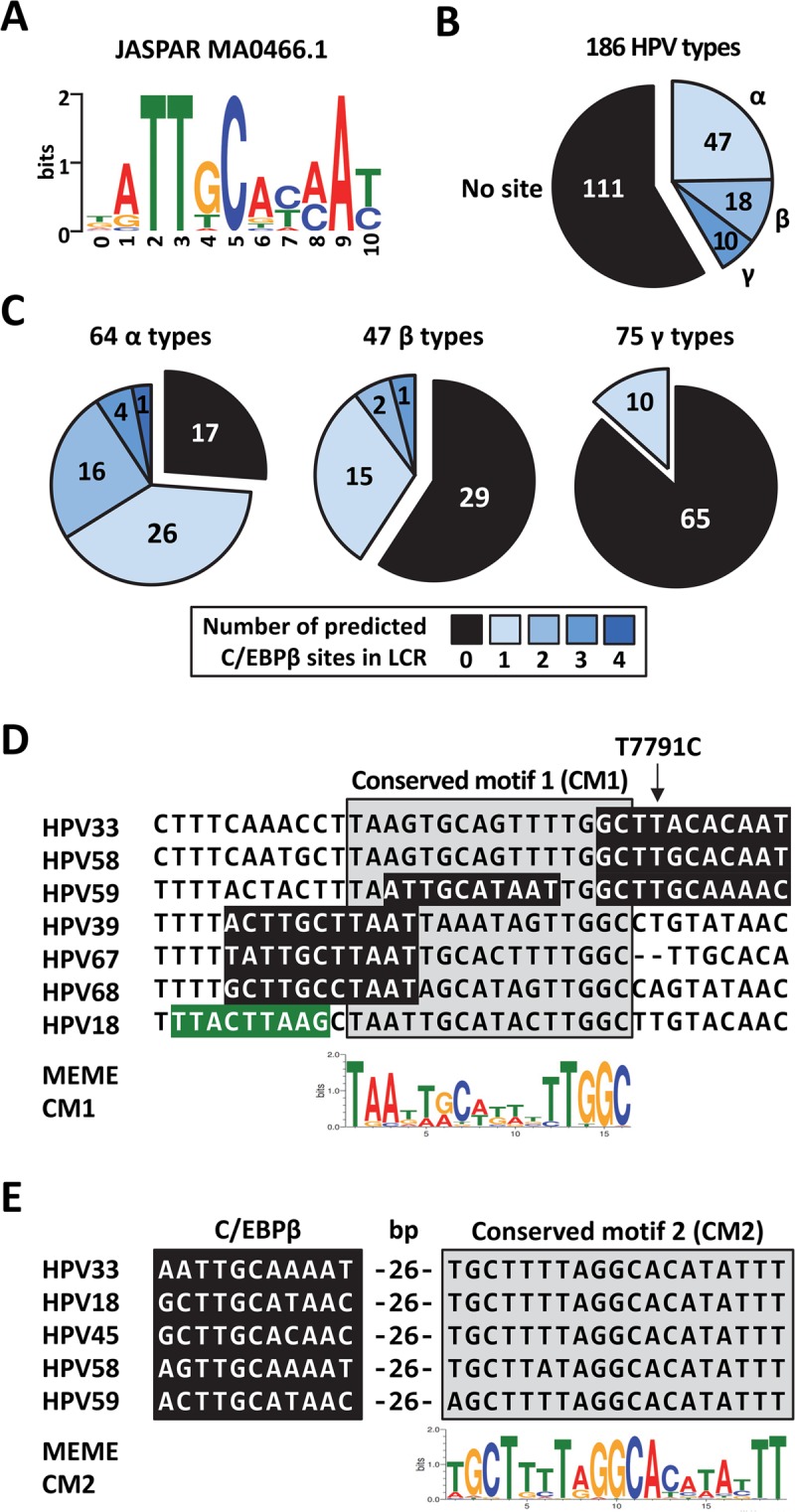
Prediction of C/EBPβ binding sites in the LCRs of 186 HPV types. (**A**) Sequence logo representation of the C/EBPβ PWM from JASPAR (MA0466.1). (**B**) Pie chart showing the number of HPV types from the α, β and γ genera that are predicted to contain one or more C/EBPβ site in their LCR. A total of 186 different HPV types were surveyed. (**C**) Pie charts showing the number of HPV types from either the α, β or γ genus, as indicated, that are predicted to contain 0, 1, 2, 3 or 4 C/EBPB site(s) in their LCR (colored according to the legend). The number of different HPV types surveyed in each genus is indicated above each chart. (**D**) Sequence alignment of the HPV33 LCR region containing BS1 with the cognate regions from the indicated HPV types. Predicted C/EBPβ sites are highlighted in black and the HPV18 switch region in green. The location of T7791C in BS1 is indicated by an arrow. Conserved motif 1 (CM1) identified from the analysis of 64 α HPV types using the MEME software is indicated by a grey box and represented by the sequence logo below the alignment. (**E**) Sequence alignment of the BS2-containing region of HPV33 and other indicated HPV types. Predicted C/EBPβ sites are highlighted in black. Conserved motif 2 (CM2) identified by the MEME software is indicated by a grey box and represented by the sequence logo below the alignment. The number of base pairs (bp) separating the predicted C/EBPβ site and CM2 is indicated for each HPV type.

To determine if regulation of the LCR by C/EBPβ is a conserved mechanism amongst HPV types, we used the JASPAR MA0466.1 PWM (Fig. 7A) to predict the occurrence of C/EBPβ binding sites in the LCRs of 186 different HPV types (available in the Papillomavirus Episteme database, PAVE, http://pave.niaid.nih.gov/ [2018])^42^. Seventy-five types (41%) were predicted to contain one or more C/EBPβ binding sites in their LCRs (Fig. 7B and Supplementary Tables S1 and S2. In terms of genera, this translated into 47 of the 64 α types surveyed (i.e. 73%) containing at least one C/EBPβ site (Fig. 7C) while only 18 of 47 β types (38%) and 10 of 75 γ types (13%) were predicted to contain one. The number of predicted C/EBPβ sites per LCR was also higher for the α types, with 21 of the 47 (45%) α LCRs containing more than one site (Fig. 7C). Thus, while regulation of the LCR by C/EBPβ appears to be a common mechanism used by α HPV types, it seems less frequently used by viruses from the β and γ genera. Comparison between high-risk and low-risk viruses revealed that C/EBPβ binding sites are overrepresented in the LCR of the high-risk HPV types 16, 18, 31, 33, 35, 39, 45, 51, 52, 56, 58, 59 and 68, with HPV31 and HPV56 being the only two lacking a putative site. As for the low-risk viruses, only 5 contained a predicted site (HPV 40, 42, 44, 61, 81) while six others were not (HPV 6, 11, 43, 53, 54, 72, 73). Thus, regulation by C/EBPβ may be more widespread amongst high-risk HPV types from the α genus.

The LCR can be divided into the 5′, central and 3′ regions, relative to the positions of the four E2 binding sites (Fig. 5A). Of a total 106 predicted C/EBPβ binding sites, 21 are found in the 5′ region, 48 in the central region, and 37 in the 3′ region (Supplementary Fig. S3). Hence, most predicted C/EBPβ sites are either in the central region, the portion of the LCR where HPV33 BS1 and BS2 are located, or in the 3′ region. Analysis of the LCR from 64 α HPV types with the MEME software identified two conserved sequence motifs located near BS1 and BS2, respectively. Figure 7D shows that BS1 overlaps conserved motif 1 (CM1) and, furthermore, that 5 other HPV types also contain a putative C/EBPβ site overlapping this motif. Interestingly, the previously described switch region in HPV18 is also close to CM1 (Fig. 7D). As for BS2, it lies 26 bp upstream of conserved motif 2 (CM2), in a region that appears to be a hotspot for C/EBPβ binding sites (Supplementary Fig. S3). Indeed, 23 HPV types contain a site in the 40 bp region upstream of CM2 or overlapping this motif (Supplementary Fig. S3). In five of these types including the highly-prevalent HPV18, the predicted C/EBPβ site is located 26 bp upstream of CM2, exactly like HPV33 BS2 (Fig. 7E). As for the predicted sites in the 3′ portion of the LCR, where the minimal origin of viral DNA replication (ori) is located, 21 out of 37 lie in a short region located immediately downstream of E2-binding site 2, which we refer to as the ori-hotspot (Supplementary Fig. S3). In summary, of the 106 predicted sites, 26 (25%) are in the BS2 hotspot, 21 (20%) in the ori-hotspot, and 7 (6%) overlap CM1. These observations suggest that the occurrence of C/EBPβ sites in the BS2- and ori-hotspots has been evolutionarily favored. In contrast, the presence of a C/EBPβ site overlapping CM1 is less common, although not unique to HPV33.

The predictions described above were performed using a cut-off p value of 0.0005 (p ≤ 0.0005). This relatively permissive p value was chosen to capture as many sites as possible and because HPV33 BS1 and BS2 are predicted at p values of 0.00021 and 0.000048, respectively (Supplementary Table S1). We also analyzed our predictions using a more stringent cut-off value of p ≤ 0.0001 to focus on “high-confidence” sites. This identified 20 sites (highlighted in black in Supplementary Fig. S3 and Supplementary Table S1) distributed among 20 HPV types. Fourteen of these sites (70%) are in the LCR of the α HPV types 18, 32, 33, 35, 45, 58, 59, 61, 62, 67, 71, 81, 89 and 90, while the other 6 (30%) are in the LCR of β types 5, 12, 36, 47, 99 and 105. High-confidence site were not found in the LCR of γ viruses. As to their location in the LCR, 3 (15%) of the high-confidence sites overlap MEME CM1, 8 (40%) are in the BS2-hotspot and 5 (25%) in the ori-hotspot. Thus, high-confidence C/EBPβ sites are also enriched in the LCR of α HPV types and are preferentially located in the BS2- and ori-hotspots and, to a lesser extent, overlapping CM1.

Collectively, the predictions described above suggest that C/EBPβ regulates the EP of several HPV types, particularly those of the high-risk viruses from the α genus. Furthermore, the fact that almost half of the predicted C/EBPβ sites lie in the BS2- and ori-hotspot regions of the LCR raises the possibility that these regulatory elements have been conserved during evolution.

### The BS2-like site in HPV18 binds C/EBPβ and activates transcription from the EP

**Figure 8:**
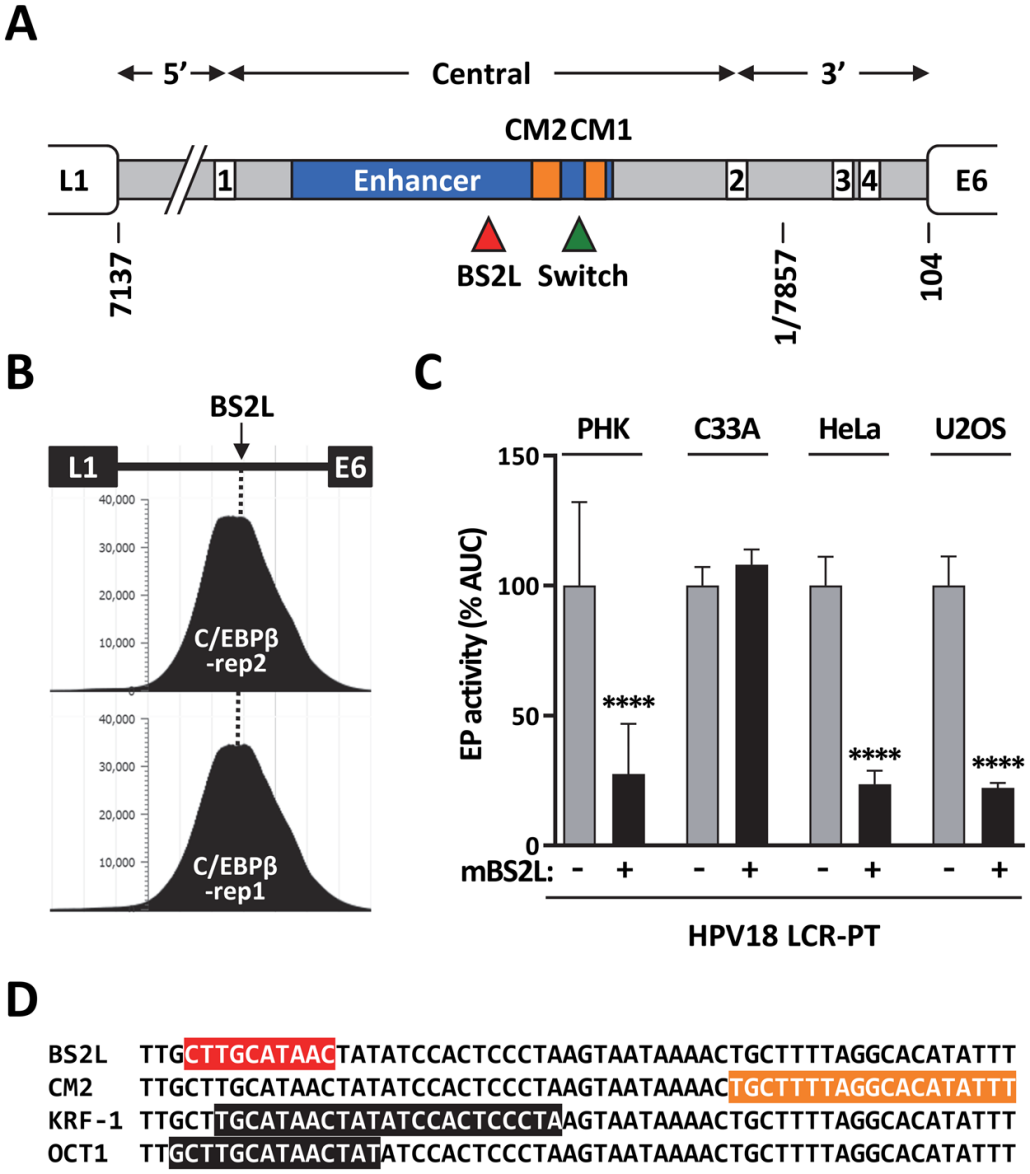
A C/EBPβ binding site similar to HPV33 BS2 activates of the HPV18 EP in PHK, HeLa and U2OS cells. (**A**) Schematic representation of the HPV18 LCR (grey box) located between the L1 and E6 ORFs. The 4 E2 binding sites are indicated by white boxes numbered from 1 to 4, and the epithelial-specific enhancer by a blue box. The C/EBPβ binding site BSL2 and the switch region are indicated by a red and a green triangle, respectively. The two MEME conserved motifs CM1 and CM2 are indicated by orange boxes. The 5′, central and 3′ regions of the LCR are delineated by double headed arrows. (**B**) Density graphs depicting the number of C/EBPβ ChIP-sequencing reads mapped to the HPV18 LCR in HeLa cells. The results obtained from two experiments (rep1 and rep2) performed by the ENCODE consortium and available from the PAVE database are shown. The LCR is represented at the top of the figure with the arrow and dotted line indicating the position of the BS2-like (BS2L) site. (**C**) Bar graphs showing the transcriptional activities of the HPV18 LCR-PT and mutant derivative carrying mBS2L, as indicated. Transcriptional activities were measured by luciferase reporter-gene assays in PHK, C33A, HeLa and U2OS cells, as indicated, and reported as described in the legend of Fig. 1 relative to the activity of LCR-PT in each cell type which was used as the reference (100%). Transcriptional activities that differ significantly from that of LCR-PT in each cell type are indicated. (**D**) Sequence of the HPV18 LCR region containing C/EBPβ BS2L, highlighted in red. Also shown are the locations of MEME CM2 (orange) and of previously described binding sites for the transcription factors KRF-1 and OCT1 (black).

The prediction that HPV18 contains a BS2-like site in its LCR, which we termed BS2L and is different than the previously identified switch region (Fig. 8A), was of interest since ChIP studies performed by the ENCODE consortium revealed that C/EBPβ is present on the enhancer region of the integrated HPV18 genome in HeLa cells (Fig. 8B). The entire ENCODE data pertaining to HPV18 in HeLa cells has been reviewed^43^ and is available from the PAVE database. This prompted us to characterize the BS2L site in HPV18 to determine if its function is similar to that of HPV33 BS2. First, we determined by fluorescence anisotropy that BS2L could bind C/EBPβ *in vitro* with an affinity (K_i_) of 154 ± 37 nM, which is only 2.3-fold higher than the affinity measured for HPV33 BS2 (Table 2). Next, we inactivated BS2L in the HPV18 LCR using a quadruple mutation (5′-CTTGCATAAC-3′ to 5′-CCCGCATCCC-3′, mutated nucleotides underlined) and measured the transcriptional activity of the resulting mutant LCR. Results presented in Fig. 8C showed that mBS2L decreased transcription from the HPV18 EP approximately 4-fold in PHK, HeLa and U2OS cells but had little to no effect in C33A cells. These results are remarkably similar to those obtained for mBS2 in the HPV33 LCR (Fig. 5), with the difference that mBS2L was slightly more deleterious in U2OS cells than mBS2. These results indicate that BS2L activates transcription from the HPV18 EP, like BS2 in HPV33. More generally, the similar activities of BS2L and HPV33 BS2 *in vitro* and *in vivo* support the idea that transactivation of the EP by C/EBPβ bound at BS2 is an evolutionarily conserved mechanism.

## Discussion

This study was spurred by the discovery that the LCR from of a rare naturally-occurring HPV33 variant, LCR10, exhibited higher transcriptional activity than other B-lineage LCRs in PHK, C33A, HeLa and U2OS cells. The T7791C variation was found to be responsible for the higher activity of LCR10 in C33A, HeLa and U2OS cells, but not in PHK where it repressed the EP in combination with T7365C and C7531T. Rather, the higher activity of LCR10 in PHK could be ascribed to the fact that it lacks the G18A variation, which is present in the other B-lineage LCRs and represses their transcriptional activity.

LCR variations can in principle affect transcription from the EP either by enhancing or inhibiting the binding of cellular transcription factors to sites present in the LCR or by creating new sites not present in the prototype. Examination of the sequence around T7791C revealed a putative binding site for the transcription factor C/EBPβ, which we termed BS1. *In vitro* experiments confirmed the binding of C/EBPβ-homodimers to BS1 and the deleterious effect of T7791C on this interaction. Moreover, mutation of BS1 (mBS1) increased the transcriptional activity of the prototype LCR in all four cell types, indicating that this site represses the EP. The stimulatory effect of T7791C and mBS1 were comparable in C33A, HeLa and U2OS cells, consistent with the idea that they affect the same regulatory element. Unlike mBS1, however, T7791C failed to activate the EP in PHK for reasons that remain unknown. It is possible that T7791C is less efficient than mBS1 at preventing the binding of C/EBPβ to BS1 because it affects only one of the two half-sites which comprise this site, in contrast to the quadruple mBS1 mutation which affects both. Additional experiments will be needed to resolve this issue.

BS1 overlaps a conserved region in the central part of the LCR which we identified as MEME CM1 (Fig. 7D). Interestingly, this region was previously shown to support the cooperative binding of the NF1 and Oct1 transcription factors *in vitro*, and to activate the EP *in vivo*^44^. This raises the possibility that the interaction of C/EBPβ with BS1 represses the EP by preventing the binding of the Oct1/NF-1 transcriptional activators to the overlapping CM1. It is intriguing that the previously identified “switch” region of HPV18 also lies near CM1 (Fig. 7D). Studies in HeLa cells suggested that a C/EBPβ-YY1 complex assembles on the switch region and activates the EP by “switching” the activity of a downstream AT-rich region from a silencing to an activating element, hence its name^14,16^. The AT-rich region is located in the 3′ part of the LCR and contains binding sites for the transcriptional repressors CUX1 (CDP/Cut)^45^ and YY1^15,46^, as well as sites for the E1 helicase which are used to initiate viral DNA replication (Supplementary Fig. S4). The C/EBPβ-YY1 complex bound at the HPV18 switch region was suggested to activate the EP by somehow converting the activity of YY1 bound at the AT-rich region from a repressor to an activator of transcription^14^. Future experiments should therefore aim at determining if BS1 in the HPV33 LCR has a similar function as the switch region in HPV18. In support of this possibility, we note that the AT-rich region of HPV33 was found to act as a transcriptional silencing element, similarly to the one in HPV18^45^. Remarkably, the G18A variation that lowers transcription from all B-lineage LCRs other than LCR10 also lies in this AT-rich region (Supplementary Fig. S4). This raises the possibility that G18A represses the EP by altering the activity of the AT-region and/or its functional interplay with BS1 if the latter functions as a switch region, although this remains to be tested.

In addition to BS1, we also identified a second C/EBPβ binding site (BS2) which, in contrast to BS1, activates the HPV33 LCR in PHK, HeLa and U2OS cells but not in C33A cells which lack the SNF/SWI ATPases BRM and BRG1, as mentioned previously. BS2 lies upstream of MEME CM2, in a region of the LCR that is predicted to be a hotspot for C/EBPβ (Supplementary Fig. S3). In support of this prediction, we have found that the BS2-like site (BS2L) in HPV18 has indeed a similar activity as HPV33 BS2 in all four cell types tested. Interestingly, BS2L overlaps a region of the LCR that was previously shown to bind the KRF-1 and OCT1 transcription factors in a mutually exclusive manner^47^ (Fig. 8D). KRF-1 was defined as an activity from HeLa cell nuclear extracts that binds to a specific site in the LCR *in vitro*^47^. Analysis of this site in reporter gene assays suggested that KRF-1 functions as an activator of the EP in PHK and in different epithelial cell lines^47,48^. Given that the precise identity of KRF-1 remains unknown, an exciting hypothesis is that C/EBPβ is part of the complex that assembles on the KRF-1 binding site to transactivate the HPV18 EP. Additional work will be needed to address this possibility.

Our predictions of C/EBP sites in the LCR of 186 HPV types made use of the JASPAR MA0466.1 PWM (Fig. 7A). Since it was generated by genome-wide ChIP studies, this PWM is arguably a superior predictive tool, with higher information content and *in vivo* relevance than the previously available C/EBPβ consensus sequence 5′-(A/G)TTGCG(C/T)AA(C/T)-3′ derived from *in vitro* studies^11^. Our own mutational analysis of the C/EBPβ 5′-ATTGC-3′ half-site confirmed the importance of the two T residues at positions 2 and 3 and of C at position 5 for high-affinity binding *in vitro*, but also indicated that C is tolerated at position 1 as found in previous *in vitro* binding site selection experiments^49^. Satisfyingly, many HPV types appear to contain a site similar to BS2 and fewer also contain a BS1-like sequence, in particular those from the α genus. This reinforces the notion that these sites have been evolutionarily conserved and thus functional. In addition to the region spanning BS2, our predictions suggested a second hotspot for C/EBPβ located in the 3′-part of the LCR between E2BS2 and the AT-rich transcriptional regulatory element (Supplementary Fig. S4). Although neither HPV33 nor HPV18 contain a site in this region, this finding is intriguing as E2 has been shown to interact with C/EBPβ and may thus help recruit it to this E2BS2-proximal site, in some HPV types^50^. To our knowledge, this hotspot region has never been identified nor investigated previously, presumably because the 25 HPV types (14 α, 9 β, and 2 γ types) that are predicted to contains a C/EBPβ site in this region are not the ones commonly studied. Our predictions revealed an over‐-representation of C/EBPβ sites in the LCR of α HPV types compared to β and γ types. This observation was unexpected but may be linked to the fact that most α HPVs infect mucosal tissue in contrast to β and γ types which reside in the skin, but this is only an hypothesis at the moment.

The fact that our studies were conducted in four cell types, PHK, C33A, HeLa and U2OS cells, has allowed us to compare our results across these different cellular models and gauge their value for the study of the HPV EP. To facilitate this comparison, we summarized all our transcriptional results from luciferase reporter-gene assays in the form a heatmap (Supplementary Fig. S5). Three main points can be made from this comparison. First, the activity of the different B-lineage variants LCRs (LCR4, 7, 8, 9 and 10) and LCR10 revertants is generally lower in PHK than in the three transformed cell lines, relative to the prototype. This is in part because these B-lineage LCRs contain the 79 del variation that reduces EP activity by ~2-fold in PHK but has no significant effect in C33A, HeLa and U2OS cells (Supplementary Fig. S1). The EP activity of LCR4, 7, 8 and 9, but not of LCR10, is also further reduced by the G18A variation which exerts its greatest inhibitory effect in PHK (Fig. 2). The second point that can be made from the heatmap is that all B-lineage LCRs and LCR10 revertants behaved similarly in the three cancer cell lines (C33A, HeLa and U2OS) although small differences were noted in the magnitude of EP stimulation by the T7791C variation. In contrast, the HPV33 and HPV18 LCRs that contain the BS2 and BS2L mutation, respectively, behaved differently in C33A cells than in the HeLa and U2OS lines. Specifically, while the BS2/BS2L mutation reduced EP activity in HeLa and to a lesser degree in U2OS cells, it had no effect in C33A cells. This illustrate the third point, which is that C33A cells appear to be defective in supporting the transactivating function of C/EBPβ bound at BS2/BS2L, presumably because these cells express little to no BRM and BRG1, as mentioned above. It is therefore apparent from these comparisons that some variations can have a cell-type specific effect (e.g. 79 del) and that caution should be exerted when considering the use of C33A cells for the study of C/EBPβ-regulated promoters such as the HPV LCR.

In conclusion, we have described here a naturally-occurring variation, T7791C, which enhances the transcriptional activity of the HPV33 EP by disrupting an inhibitory C/EBPβ binding site. A second C/EBPβ element was also identified that has a functional counterpart in HPV18 and possibly in many other HPV types predicted to contain a similar site. Taken together, our findings suggest that C/EBPβ can both activate and repress transcription of the HPV33 early genes via two separate binding sites and that it can also regulate the EP of several other HPV types mostly from the α genus.

## Methods

### HPV33 LCR variants

The HPV33 LCR10 variant (accession number KT827350) was previously identified from a cervical sample of a woman co-infected with the human immunodeficiency virus (HIV)^29,30^. In this study, LCR10 was amplified by PCR from the same cervical sample and sequenced in two independent experiments exactly as described previously for LCR4, LCR7, LCR8 and LCR9^28–30^. Cervical samples were obtained specifically for research purposes and processed as described previously^29,30^. All participants provided written informed consent. The Research and ethics committees of the Centre Hospitalier de l’Université de Montréal approved the study. All experiments were performed in accordance with relevant guidelines and regulations. Nucleotide sequence variations are reported according to the numbering scheme of the HPV33 reference genome (GenBank accession M12732.1). All LCRs contained the A81C transversion found in other HPV33 isolates; a C at position 81 is the correct nucleotide although it is mistakenly replaced by an A in the GenBank reference sequence.

### Plasmid construction and mutagenesis

The plasmids expressing Renilla luciferase (Rluc) from the prototypical HPV33 LCR (LCR-PT) or from the LCR4, LCR7, LCR8 and LCR9 variants, as well as the internal control plasmid encoding firefly luciferase (Fluc) downstream of LCR-PT (LCR-PT-Fluc) have been described previously^28^. An identical cloning strategy was used to construct the plasmids expressing Rluc from LCR10 and from the HPV18 prototype LCR (nt 7137–104 in the HPV18REF sequence from PAVE, a revised version of the original GenBank X05015.1 sequence). Mutations and reversions in the HPV33 and HPV18 LCRs were created using the QuikChange Site-Directed Mutagenesis kit (Stratagene). Plasmids used for bacterial expression of rat C/EBPβ fragments fused to glutathione-S-transferase (GST) were constructed by inserting an EcoRI/SalI-digested PCR product encoding either the basic region alone (B, amino acids 262–298) or the basic region and leucine zipper domain (bZIP, amino acids 262–345) into the pGEX-4T1 vector. All constructs were verified by DNA sequencing. Additional information on the construction of these plasmids will be made available upon request.

### Cell culture and transfection

PHK of epithelial origin were purchased from Cell Applications Inc. and grown in EpiVita Adult Keratinocytes Growth Medium (Cat. No. 141–500a, Cell Applications Inc.) as recommended by the manufacturer. The C33A, HeLa and U2OS cell lines were grown in Dulbecco’s Modification of Eagle’s Medium (DMEM) supplemented with 10% fetal bovine serum (FBS), 50 I.U. of penicillin/ml, 50 µg of streptomycin/ml and 2 mM of l-glutamine (Wisent Bioproducts). PHK were transfected with the Cytofect-Epithelial Cell Transfection Kit (cat. No. TF102K, Cell Applications Inc.) while C33A, HeLa and U2OS cells were transfected using the Lipofectamine 2000 reagent (Cat. No. 11668–500, Life Technologies) according to the manufacturer’s recommendations.

### Luciferase-reporter gene assay

This assay has been described previously^28^. Briefly, PHK, C33A, HeLa and U2OS cells were plated in white flat-bottom 96-well plates (Cat. No. 3917, Corning) at a density of 10 000 (PHK), 15 000 (HeLa) and 25000 (C33A, U2OS) cells/well 24 h prior to transfection. Cells were transfected with increasing amounts of each LCR-Rluc plasmid (25, 50, 100 and 200 ng) and a fixed amount of LCR-PT-Fluc (25 ng) as an internal control. The pGL3-Rluc plasmid (200 ng) was used as a negative control. Both Fluc and Rluc activities were measured with the Dual-Glo luciferase assay system and a GloMaxTM 96-well luminometer (Promega) 24 h post transfection. The Rluc/Fluc ratios determined at increasing amounts of each LCR-Rluc plasmid were used to calculate an area-under-the-curve (AUC) value that is reported as an overall activity of each LCR. The EP activity of each LCR-Rluc plasmid was tested in triplicates in at least 2 independent experiments in PHK, and in duplicates in at least 4 independent experiments in C33A, HeLa and U2OS cells. Statistical significance was tested by ANOVA with Dunnett’s post-hoc analysis.

In C/EBPβ overexpression studies, 100 ng of LCR-PT plasmid was co-transfected into cells with a rat C/EBPβ-expression plasmid encoding either the LAP (5 ng) or LIP (10 ng) isoform (kindly provided by Janice Clements via Lou Laimins). The internal control plasmid used in these experiments was LCR-ΔC/EBPβ-Fluc, a derivative of LCR-PT-Fluc which contains the mBS1 and mBS2 mutations as well as an additional mutation (ATTGTACAAT changed to ACCGTACCCT, nt 7471–7480 in GenBank M12732.1) in a third possible C/EBPβ binding site that was neither predicted by the JASPAR MA0466.1 PWM nor was inactive *in vivo* (data not shown). LCR-ΔC/EBPβ-Fluc was used instead of LCR-PT-Fluc in an attempt to mitigate any possible effect of LAP or LIP on the internal control plasmid. All other steps were performed as described above.

### Western blot and antibodies

Cells were resuspended in lysis buffer (50 mM Tris pH 8.0, 150 mM NaCl, 1% Triton X-100 with protease inhibitors [2 ug/ml Leupeptin, 1 ug/ml Apoprotinin, 1 ug/ml Petpstatin a, 0.1 mM PMSF]) and 20 μg of whole cell extract subjected to SDS-PAGE. C/EBPβ LAP and LIP isoforms were detected using a rabbit polyclonal antibody (Santa Cruz Biotechnology, cat: sc-150) and a horseradish peroxidase-conjugated sheep anti-rabbit antibody (GE healthcare, cat: NA934). Β-tubulin was detected using a mouse monoclonal antibody (Sigma-Aldrich, cat: T4026) and a horseradish peroxidase-conjugated sheep anti-mouse antibody (GE healthcare, cat: NA931). All proteins were detected using an enhanced chemiluminescence detection kit (GE Healthcare).

### Recombinant C/EBPβ expression and purification

GST-C/EBPβ fusion proteins were expressed and purified as previously described^28^ with the following modifications. Briefly, bacterial cultures of *E*. *coli* BL21 cells (Novagen) transformed with the GST, GST-C/EBPβ-b and GST-C/EBPβ-bZIP expression plasmids were grown in Luria-Bertani medium to an optical density of 0.5 at 595 nm and induced for 4 hours by the addition of 0.5 mM of isopropyl-β-D-thiogalactopyranoside. Cells harvested from a 2-liter culture were resuspended in 30 mL of lysis buffer (50 mM Tris-HCl [pH 7.6], 1 M NaCl, 5 mM EDTA, 5 mM DTT, 10% glycerol, 0.1% NP-40, 10 ug/mL antipain, 2 ug/mL leupeptin, 1 ug/mL pepstatin A, 2 ug/mL aprotinin and 1 mM phenylmethanesulfonylfluoride). Cells were disrupted by sonication and lysates were cleared by centrifugation at 9000 g for 20 minutes and incubated with 2 mL of glutathione-Sepharose 4B beads (GE Healthcare, cat: 17-0756-01) for 3 hours at 4 °C. Beads were washed with 15 bed volumes of high-salt buffer (50 mM Tris-HCl [pH 8.0], 0.5 M NaCl, 5 mM EDTA, 5 mM DTT, 10% glycerol, 0.1% NP-40) followed by 15 bed volumes of low-salt buffer (0.2 M NaCl). Proteins were eluted in elution buffer (25 mM Tris-HCl [pH 8.0], 0.2 M NaCl, 1 mM EDTA, 5 mM DTT, 10% glycerol, 0.1% NP-40, 0.01 M reduced glutathione) and recovered after centrifugation at 5000 g for 5 minutes. Protein concentrations were determined by Bradford analysis.

### Fluorescence anisotropy DNA-binding assay

Binding reactions were performed in OptiPlate-96F high-binding-affinity black 96-well plates (Perkin Elmer) in a final volume of 150 μL of assay buffer (25 mM Tris-HCl [pH 7.6], 0.1 mM DTT, 0.01% NP-40, 75 mM NaCl, 150 mM KCl and 5 mM MgCl_2_) containing 15 nM of fluorescein-labeled BS1 probe and the indicated concentration of purified GST, GST-C/EBPβ-b or GST-C/EBPβ-bZIP protein. Competition binding assays were performed similarly using a final concentration of 15 nM of BS1 probe, 125 nM of GST-C/EBPβ-bZIP protein and increasing amount of unlabeled competitor DNA (0, 5.9, 11.7, 23.4, 46.9, 93.8, 187.5, 375, 750 and 1500 nM). Fluorescence readings were taken after 20 minutes of incubation at room temperature with a Victor^3^V multilabel plate-reader (Perkin Elmer) equipped with a 485-nm/535-nm filter set. The background fluorescence of the probe was subtracted from all values.

The fluorescein-labeled BS1 probe with the fluorophore attached at the 5′ end by a six-carbon linker and the unlabeled oligonucleotides were purchased from Integrated DNA Technologies (IDT). The sequence of the BS1 probe was as follows: 5′-TTTGGCTTACACAATTGCTTT-3′ (C/EBPβ BS1 underlined). All competitor oligonucleotides described in Table 2 contained variations of the C/EBPβ consensus site in the context of the following sequence: 5′-CCCTGATTGCGCAATAGGCTC-3′ (consensus site underlined). The sequences of the HPV18 BS2L oligonucleotide was as follows: 5′-CCCTGCTTGCATAACAGGCTC-3′ (BS2L underlined). The duplex fluorescein-labeled probe and duplex unlabeled competitor DNAs were prepared by annealing complementary oligonucleotides at a ratio of 1:1.5 and of 1:1, respectively. The mixture was heated at 95 °C for 5 minutes in annealing buffer (25 mM Tris-HCl [pH 7.6], 1 mM DTT, 0.1 M NaCl, 0.01% NP-40) and allowed to cool slowly to room temperature as previously described^51^.

### K_D_ and K_i_ measurements

K_D_ and K_i_ values were obtained by fitting the data from direct binding isotherms and from inhibitory dose-response curves, respectively, with nonlinear least-squares regression to a standard equation describing a single binding-site equilibrium, using GraphPad Prism version 7.03, as described previously^51^. Values were obtained from three independent experiments with each data point performed in duplicate. No corrections for emission intensity were performed since the quantum yield of the fluorescent probes did not change significantly upon C/EBPβ binding.

### Bioinformatic prediction of C/EBPβ binding sites

The LCR sequences from 186 different HPV types were downloaded from the Papillomavirus Episteme database (PAVE, http://pave.niaid.nih.gov/ [2018])^42^. Putative C/EBPβ binding sites were predicted with the FIMO software of the MEME-Suite^52^ using the MA0466.1 PWM from the JASPAR database^36^ and a cut-off p value p ≤ 0.0005, or of p ≤ 0.0005 to identify “high-confidence” sites.

### Statistical analysis

Statistics and AUC calculations and the graphical representation of the data in the form of a heatmap were all using GraphPad Prism version 7.03.

## Supplementary information

Supplementary information

## Data Availability

The datasets generated during and/or analyzed during the current study are available from the corresponding author on reasonable request.
