# Supplementary material for: Characterization of an HPV33 natural variant with enhanced transcriptional activity suggests a role for C/EBPβ in the regulation of the viral early promoter
Source: Sci Rep. 2019 Mar 25;9:5113. doi: 10.1038/s41598-019-41102-7 (PMC6433916; doi:10.1038/s41598-019-41102-7)
Supplement: Supplementary file 1 — Supplementary information [file 41598_2019_41102_MOESM1_ESM.pdf]

Supplementary Information

Characterization of an HPV33 natural variant with enhanced transcriptional activity suggests a role for C/EBPβ in the regulation of the viral early promoter

Jennifer Alvarez [1,3,4], David Gagnon [1,3], François Coutlée [5], Jacques Archambault [1,2,3,4,*]

[1] Department of Microbiology and Immunology, McGill University, Montreal, QC, Canada

[2] Division of Experimental Medicine, McGill University, Montreal, QC, Canada

[3] Institut de recherches cliniques de Montréal (IRCM), Montreal, QC, Canada

[4] Department of Biochemistry and Molecular Medicine, Université de Montréal, Montreal, QC, Canada

[5] Centre de Recherche et Département de Microbiologie Médicale et Infectiologie, Centre Hospitalier de l'Université de Montréal (CHUM), Université de Montréal, Montréal, QC, Canada

* Corresponding author: Jacques Archambault, Department of Microbiology, McGill University, Montreal, QC, Canada. Email: jacques.archambault2@mcgill.ca

Table of Content

- Supplementary Figure S1 page 2
- Supplementary Figure S2 page 3
- Supplementary Figure S3
  - Figure and legend page 4
  - High-resolution figure page 5
- Supplementary Figure S4 page 6
- Supplementary Figure S5 page 7
- Supplementary Table S1 pages 8 and 9
- Supplementary Table S2 page 10
- Supplementary references page 11

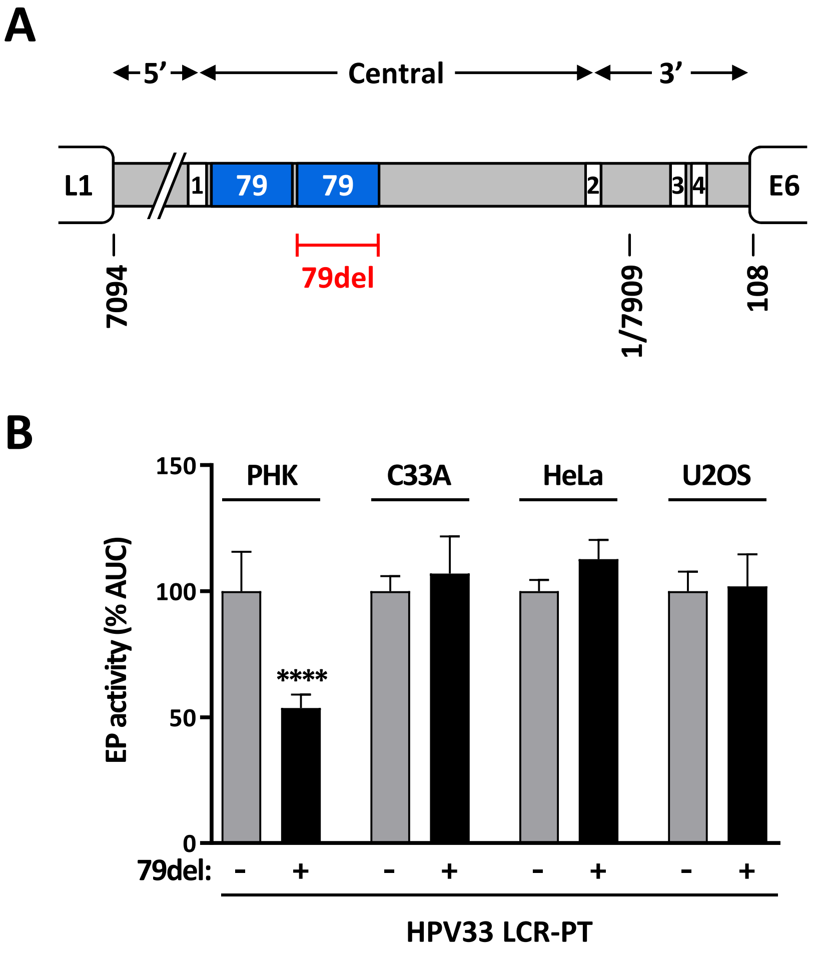

Supplementary Figure S1. Effect of the 79del variation on the activity of the HPV33 EP. (A) Schematic representation of the HPV33 LCR (grey box) located between the L1 and E6 ORFs. The region removed by the 79del variation is indicated in red. The 4 E2 binding sites are indicated by white boxes numbered from 1 to 4. The 5', central and 3' regions of the LCR are delineated by double headed arrows. (B) Bar graphs showing the transcriptional activities of LCR-PT and of the mutant derivative carrying the 79del variation in PHK, C33A, HeLa and U2OS cells, as indicated, and reported relative to the activity of LCR-PT in each cell type which was used as the reference (100%). Transcriptional activities that differ significantly from that of LCR-PT in each cell type are indicated. Note that the data obtained in PHK and HeLa cells has been previously published in Alvarez et al.[1]

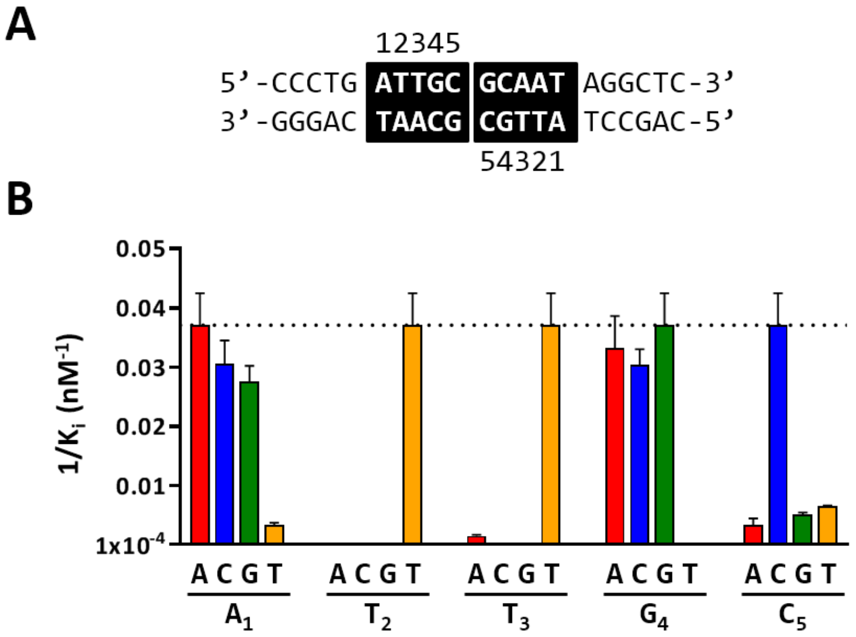

Supplementary Figure S2. Systematic mutagenesis of the C/EBPβ consensus binding sequence. (A) Sequence of the C/EBPβ consensus oligonucleotide that was used for mutagenesis. The two 5’-ATTGC-3’ inverted half sites are highlighted in black with each nucleotide position numbered from 1 to 5, as indicated. Each position was mutated to the other three possible nucleotides, in both half sites simultaneously. For example, mutation of position 1 from A to T (A_1 to T) yielded a double-mutant oligonucleotide of the sequence 5’-<u>T</u>TTGCGCAA<u>A</u>-3’ (mutations underlined). Mutant oligonucleotides were used as competitors in the anisotropy GST-C/EBPβ-BZ DNA-binding assay and their K_i values measured as described in the legend of Fig. 4 and in the Methods section. (B) Bar graph showing the $1/K_i$ values of oligonucleotides in which each position of the two half-sites were mutated simultaneously to the other 3 possible nucleotides. The results show that nucleotides T_2, T_3 and C_5 could not be changed without a loss in binding-affinity, confirming their importance for interaction with GST-C/EBPβ-BZ. In contrast, nucleotides A_1 and G_4 were more permissive, with T being the only deleterious nucleotide at both positions. Collectively, these results suggest that GST-C/EBPβ-BZ binds with higher affinity to sites in which at least one of the two half sites contains T at positions 2 and 3, and C at position 5.

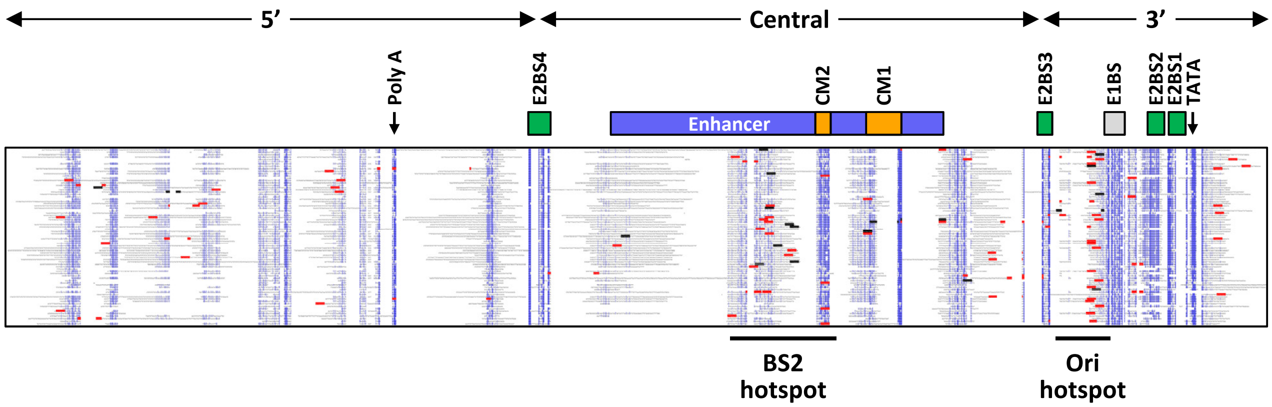

Supplementary Figure S3. Alignment of 75 HPV LCRs predicted to contain at least one C/EBPβ binding site. LCR sequences were aligned and visualized with the MUSCLE[2] and Jalview[3] software, respectively. Sequence conservation is shown using increasing intensities of blue shading. Predicted C/EBPβ binding sites are highlighted either in black for the high-confidence sites ($p<0.0001$) or in red for the remaining sites (p value between 0.0001 and 0.0005). The 5’, central and 3’ regions of the LCR are delineated at the top of the figure. Also indicated are the locations of the Late polyadenylation signal (Poly A, arrow), E2 binding sites 1 to 4 (E2BS1-4, green boxes), epithelial-specific enhancer (Enhancer, blue box), MEME conserved motifs 1 and 2 (CM1, CM2; orange boxes), E1 binding sites (E1BS, grey box) and TATA box (arrow). Two hotspot regions enriched in C/EBPβ binding sites are indicated in red at the bottom of the figure; one region spans BS2 (BS2 hotspot) while the other is located within the minimal origin of DNA replication (ori hotspot).

Note: a high-resolution version of the sequence alignment is provided on the next page.

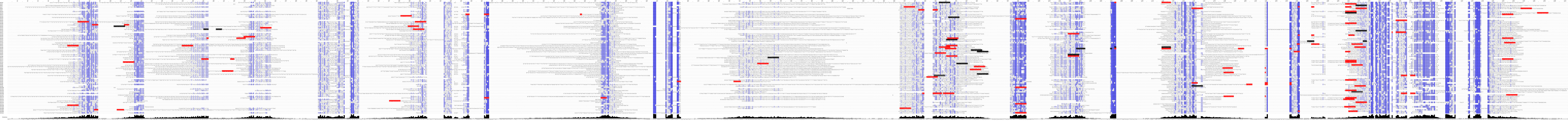

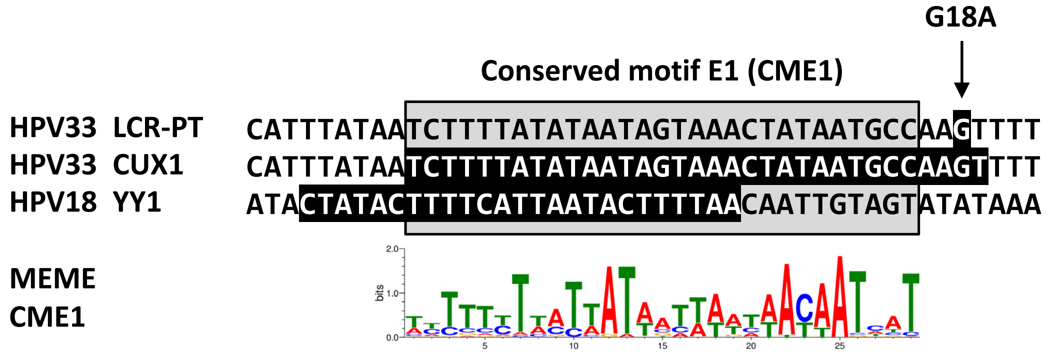

Supplementary Figure S4. Region of the LCR affected by the G18A variation. The sequence of the HPV33 prototype LCR (LCR-PT) that contains G18A (arrow) is shown at the top of the figure. A MEME conserved motif that spans the E1-binding sites in the origin (CME1) is shown by a grey box. The region of HPV33 that has been shown to bind the transcription factor CUX1 (also known as CDP/CUT) is highlighted in black[4]. The region of HPV18 that has been shown to bind the transcription factor YY1 is highlighted in black[5]. The sequence logo of MEME CME1 that was derived from the LCRs of 64 different α HPV types is shown at the bottom of the figure.

Virus	Group	LCR	BS1	BS2	T7365C	C7531T	T7791C	G18A	79del	Others	Total
HPV33	Natural LCR	LCR-PT	-	-	-	-	-	-	-	0	0
HPV33	Natural LCR	LCR4	-	-	-	-	-	+	+	16	17
HPV33	Natural LCR	LCR7	-	-	-	-	-	+	+	16	17
HPV33	Natural LCR	LCR8	-	-	-	-	-	+	+	17	18
HPV33	Natural LCR	LCR9	-	-	-	-	-	+	+	17	18
HPV33	Natural LCR	LCR10	-	-	+	+	+	-	+	16	19
HPV33	LCR10 revertant	Rev T7365C	-	-	-	+	+	-	+	16	18
HPV33	LCR10 revertant	Rev C7531T	-	-	+	-	+	-	+	16	18
HPV33	LCR10 revertant	Rev T7791C	-	-	+	+	-	-	+	16	18
HPV33	LCR10 revertant	Rev T7365C/C7531T	-	-	-	-	+	-	+	16	17
HPV33	LCR10 revertant	Rev T7365C/T7791C	-	-	-	+	-	-	+	16	17
HPV33	LCR10 revertant	Rev C7531T/T7791C	-	-	+	-	-	-	+	16	17
HPV33	LCR10 revertant	Rev T7365C/C7531T/T7791C	-	-	-	-	-	-	+	16	16
HPV33	LCR-PT mutant	79del	-	-	-	-	-	-	+	0	1
HPV33	LCR-PT mutant	T7791C	-	-			+	-	-	0	1
HPV33	LCR-PT mutant	BS1	+	-	-	-	-	-	-	0	1
HPV33	LCR-PT mutant	BS2	-	+	-	-	-	-	-	0	1
HPV33	LCR-PT mutant	BS1/BS2	+	+	-	-	-	-	-	0	2
HPV18	LCR	LCR-PT	-	-	-	-	-	-	-	0	0
HPV18	LCR	LCR-PT BS2L	-	+	-	-	-	-	-	0	1

Column group for BS1 through Total: Variations / Mutations

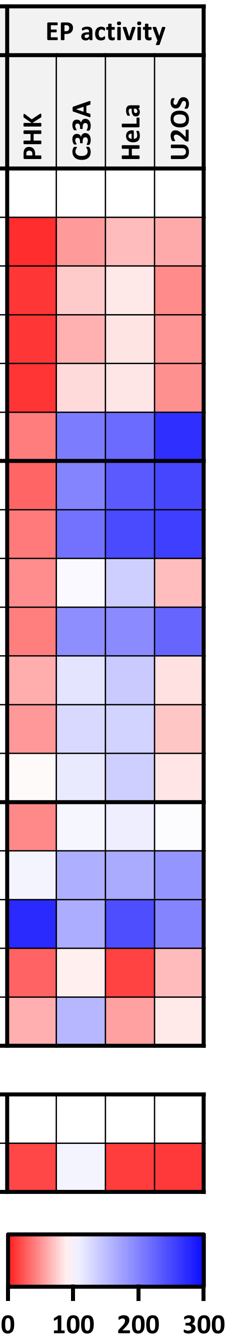

Supplementary Figure S5. Transcriptional activities of HPV variant and mutant LCRs. EP activities of the indicated LCRs are summarized in the form of a heatmap relative to the activity of the prototype (LCR-PT) which is assigned a reference value of 100% and colored in white in all four cell types. EP activity values lower and higher than 100% are colored with proportional intensities of red and blue, respectively, according to the legend shown at the bottom of the figure. Variations present and absent in each LCR are indicated by a plus sign on a black background (+) and by a minus sign (-), respectively. The number of additional variations (Others) and total number of variations in each LCR are indicated.

HPV type	Genus	p-value	q-value	Sequence	Strand	Start	End
3	Alpha	3.86E-04	0.798	AGTTGCTACAC	+	538	548
5	Beta	9.02E-06	0.428	GATTTCACAAT	+	321	331
7	Alpha	2.60E-04	0.798	GGTTGCTACAC	+	679	689
7	Alpha	2.97E-04	0.798	GGTTGCCACAC	+	494	504
8	Beta	1.19E-04	0.734	AATTGCACAAG	-	433	443
8	Beta	1.98E-04	0.734	AATTTCTTAAT	+	298	308
8	Beta	4.74E-04	0.798	AGTTTCATCAG	+	267	277
9	Beta	1.35E-04	0.734	AATTGCACCAG	-	133	143
10	Alpha	3.86E-04	0.798	AGTTGCTACAC	+	557	567
12	Beta	9.50E-05	0.734	GATTTCCCAAT	+	295	305
12	Beta	3.17E-04	0.798	CATTGCAAAAG	-	453	463
13	Alpha	1.27E-04	0.734	GCTTGCTCAAT	-	724	734
13	Alpha	4.26E-04	0.798	AGTTACAAAAT	-	228	238
16	Alpha	2.03E-04	0.734	TGTTTCAACAC	+	176	186
18	Alpha	4.99E-05	0.626	GCTTGCATAAC	+	508	518
22	Beta	3.56E-04	0.798	GGTTGCTCCAG	-	166	176
24	Beta	4.38E-04	0.798	CATTACCTAAC	+	314	324
26	Alpha	2.92E-04	0.798	TATTACACAAG	+	276	286
26	Alpha	4.31E-04	0.798	CCTTACATAAC	-	38	48
28	Alpha	3.86E-04	0.798	AGTTGCTACAC	+	680	690
29	Alpha	1.33E-04	0.734	CATTACACAAC	-	25	35
32	Alpha	3.36E-05	0.507	AGTTGCACAAC	-	22	32
32	Alpha	3.21E-04	0.798	GCTTGCTAAAT	-	194	204
33	Alpha	4.75E-05	0.626	AATTGCAAAAT	+	606	616
33	Alpha	2.10E-04	0.734	GCTTACACAAT	+	695	705
34	Alpha	1.77E-04	0.734	CATTACATAAC	-	82	92
35	Alpha	5.93E-06	0.428	CATTGCACAAT	-	114	124
35	Alpha	3.82E-04	0.798	ACTTACATAAT	+	245	255
36	Beta	3.36E-05	0.507	AATTTCACAAT	+	319	329
37	Beta	3.47E-04	0.798	TCTTTCTTCAT	+	300	310
39	Alpha	2.80E-04	0.798	ACTTGCTTAAT	+	531	541
40	Alpha	2.24E-04	0.751	GGTTGCTACAT	+	580	590
42	Alpha	2.74E-04	0.798	TGTTTCCTAAC	+	165	175
44	Alpha	1.33E-04	0.734	CATTACACAAC	-	84	94
44	Alpha	1.64E-04	0.734	GCTTGCTTAAT	-	723	733
44	Alpha	4.01E-04	0.798	AGTTGCCAAAC	-	423	433
45	Alpha	3.36E-05	0.507	GCTTGCACAAC	+	493	503
47	Beta	3.54E-05	0.507	CATTTCACAAT	+	320	330
48	Gamma	4.47E-04	0.798	GATTTCTTCAG	+	442	452
51	Alpha	1.35E-04	0.734	GCTTGCAACAT	+	746	756
51	Alpha	4.35E-04	0.798	TGTTACCTAAC	+	716	726
52	Alpha	1.28E-04	0.734	CATTGCCTAAC	+	583	593
52	Alpha	1.69E-04	0.734	AATTACATAAC	-	79	89
52	Alpha	3.76E-04	0.798	AATTACCCAAC	-	10	20
58	Alpha	2.56E-05	0.507	GCTTGCACAAT	+	564	574
58	Alpha	1.19E-04	0.734	AGTTGCAAAAT	+	476	486
58	Alpha	1.67E-04	0.734	TGTTTCAACAT	+	664	674
59	Alpha	9.47E-06	0.428	AATTGCATAAT	+	581	591
59	Alpha	1.05E-04	0.734	GGTTGCTTAAC	-	670	680
59	Alpha	1.16E-04	0.734	ACTTGCATAAC	+	506	516
59	Alpha	1.46E-04	0.734	GCTTGCAAAAC	+	594	604
61	Alpha	7.49E-06	0.428	AATTGCACAAC	-	520	530
62	Alpha	1.49E-05	0.428	TATTGCAAAAT	-	521	531

Supplementary Table S1. List of predicted C/EBPβ binding sites. Indicated for each site are the HPV type and genus in which it was predicted, the p- and q-values calculated by the FIMO software (http://meme-suite.org/tools/fimo [2018]), its sequence and the DNA strand on which this sequence is found, and its position within the LCR (Start and End) numbered relative to the first nucleotide of the LCR which is designated as position 1 (i.e. position 1 of each LCR corresponds to the nucleotide immediately 3' of the L1 STOP codon). High-confidence sites ($p<0.0001$) are highlighted in black. **Table S1 continues on next page.**

HPV type	Genus	p-value	q-value	Sequence	Strand	Start	End
62	Alpha	1.09E-04	0.734	CATTGCCCCAC	-	668	678
65	Gamma	2.04E-04	0.734	TCTTACACAAT	-	285	295
67	Alpha	2.88E-05	0.507	TATTGCTTAAT	+	517	527
68	Alpha	1.98E-04	0.734	GCTTGCCTAAT	+	571	581
68	Alpha	2.74E-04	0.798	GGTTGCCAAAC	-	502	512
71	Alpha	3.54E-05	0.507	TATTGCTTCAT	+	404	414
74	Alpha	1.33E-04	0.734	CATTACACAAC	-	124	134
74	Alpha	1.64E-04	0.734	GCTTGCTTAAT	-	770	780
77	Alpha	1.46E-04	0.734	GGTTGCCTCAC	+	558	568
78	Alpha	3.56E-04	0.798	TGTTACAACAC	-	30	40
78	Alpha	3.86E-04	0.798	AGTTGCTACAC	+	547	557
81	Alpha	1.49E-05	0.428	TATTGCAAAAT	-	534	544
81	Alpha	2.93E-04	0.798	CATTGCCACAC	+	386	396
82	Alpha	1.35E-04	0.734	GCTTGCAACAT	+	768	778
82	Alpha	3.77E-04	0.798	TGTTACCCAAC	+	738	748
83	Alpha	1.88E-04	0.734	TATTGCAAAAG	-	567	577
84	Alpha	4.78E-04	0.798	TGTTACATAAG	-	620	630
85	Alpha	3.92E-04	0.798	CCTTACATAAT	+	507	517
86	Alpha	1.33E-04	0.734	CATTACACAAC	-	110	120
86	Alpha	4.83E-04	0.798	GGTTACATAAG	-	648	658
87	Alpha	4.78E-04	0.798	TGTTACATAAG	-	672	682
89	Alpha	1.49E-05	0.428	TATTGCAAAAT	-	529	539
90	Alpha	8.96E-05	0.734	TCTTTCACAAT	+	407	417
90	Alpha	1.67E-04	0.734	TATTACAAAAT	+	640	650
91	Alpha	4.08E-04	0.798	CGTTGCCAAAC	-	335	345
93	Beta	2.12E-04	0.734	TATTACTCAAC	+	313	323
94	Alpha	3.86E-04	0.798	AGTTGCTACAC	+	556	566
95	Gamma	4.78E-04	0.798	TCTTACTTAAT	-	209	219
96	Beta	2.79E-04	0.798	AGTTTCGCAAC	+	259	269
97	Alpha	2.60E-04	0.798	GGTTGCTACAC	-	679	689
99	Beta	9.17E-05	0.734	AGTTTCACAAT	+	288	298
99	Beta	1.98E-04	0.734	AATTTCTTAAT	+	317	327
100	Beta	3.56E-04	0.798	GGTTGCTCCAG	-	174	184
101	Gamma	1.52E-04	0.734	CGTTGCAAAAC	-	305	315
103	Gamma	2.03E-04	0.734	TATTACAAAAC	-	410	420
105	Beta	7.32E-05	0.734	TATTTCTCAAT	+	300	310
106	Alpha	1.67E-04	0.734	TATTACAAAAT	+	695	705
106	Alpha	2.06E-04	0.734	CATTTCTTAAT	+	462	472
106	Alpha	4.08E-04	0.798	CATTTCCAAAT	+	450	460
107	Beta	2.03E-04	0.734	GGTTGCTAAAT	+	423	433
114	Alpha	4.78E-04	0.798	TGTTACATAAG	-	699	709
117	Alpha	3.62E-04	0.798	CATTACTTAAT	-	181	191
117	Alpha	3.86E-04	0.798	AGTTGCTACAC	+	545	555
125	Alpha	2.60E-04	0.798	GGTTGCTACAC	+	541	551
137	Gamma	4.07E-04	0.798	TGTTACCCCAC	+	96	106
143	Beta	3.38E-04	0.798	AATTACTCAAC	+	305	315
151	Beta	3.56E-04	0.798	GGTTGCTCCAG	-	166	176
158	Gamma	1.71E-04	0.734	CATTTCAACAT	-	3	13
160	Alpha	3.47E-04	0.798	AGTTGCTACAT	+	519	529
180	Gamma	3.67E-04	0.798	TGTTTCTAAAT	-	310	320
202	Gamma	1.67E-04	0.734	TATTACAAAAT	+	60	70
209	Beta	2.24E-04	0.751	GGTTGCTACAT	+	284	294
215	Gamma	1.26E-04	0.734	CGTTGCAAAAT	-	304	314

Supplementary Table S1. List of predicted C/EBPβ binding sites. Indicated for each site are the HPV type and genus in which it was predicted, the p- and q-values calculated by the FIMO software (http://meme-suite.org/tools/fimo [2018]), its sequence and the DNA strand on which this sequence is found, and its position within the LCR (Start and End) numbered relative to the first nucleotide of the LCR which is designated as position 1 (i.e. position 1 of each LCR corresponds to the nucleotide immediately 3' of the L1 STOP codon). High-confidence sites ($p<0.0001$) are highlighted in black.

HPV type	Genus	Predicted C/EBPβ sites
2	Alpha	0
3	Alpha	1
4	Gamma	0
5	Beta	1
6	Alpha	0
7	Alpha	2
8	Beta	3
9	Beta	1
10	Alpha	1
11	Alpha	0
12	Beta	2
13	Alpha	2
14	Beta	0
15	Beta	0
16	Alpha	1
17	Beta	0
18	Alpha	1
19	Beta	0
20	Beta	0
21	Beta	0
22	Beta	1
23	Beta	0
24	Beta	1
25	Beta	0
26	Alpha	2
27	Alpha	0
28	Alpha	1
29	Alpha	1
30	Alpha	0
31	Alpha	0
32	Alpha	2
33	Alpha	2
34	Alpha	1
35	Alpha	2
36	Beta	1
37	Beta	1
38	Beta	0
39	Alpha	1
40	Alpha	1
42	Alpha	1
43	Alpha	0
44	Alpha	3
45	Alpha	1
47	Beta	1
48	Gamma	1
49	Beta	0
50	Gamma	0
51	Alpha	2
52	Alpha	3
53	Alpha	0
54	Alpha	0
56	Alpha	0
57	Alpha	0
58	Alpha	3
59	Alpha	4
60	Gamma	0
61	Alpha	1
62	Alpha	2
65	Gamma	1
66	Alpha	0
67	Alpha	1
68	Alpha	2

HPV type	Genus	Predicted C/EBPβ sites
69	Alpha	0
70	Alpha	0
71	Alpha	1
72	Alpha	0
73	Alpha	0
74	Alpha	2
75	Beta	0
76	Beta	0
77	Alpha	1
78	Alpha	2
80	Beta	0
81	Alpha	2
82	Alpha	2
83	Alpha	1
84	Alpha	1
85	Alpha	1
86	Alpha	2
87	Alpha	1
88	Gamma	0
89	Alpha	1
90	Alpha	2
91	Alpha	1
92	Beta	0
93	Beta	1
94	Alpha	1
95	Gamma	1
96	Beta	1
97	Alpha	1
98	Beta	0
99	Beta	2
100	Beta	1
101	Gamma	1
102	Alpha	0
103	Gamma	1
104	Beta	0
105	Beta	1
106	Alpha	3
107	Beta	1
108	Gamma	0
109	Gamma	0
110	Beta	0
111	Beta	0
112	Gamma	0
113	Beta	0
114	Alpha	1
115	Beta	0
116	Gamma	0
117	Alpha	2
118	Beta	0
119	Gamma	0
120	Beta	0
121	Gamma	0
122	Beta	0
123	Gamma	0
124	Beta	0
125	Alpha	1
126	Gamma	0
127	Gamma	0
128	Gamma	0
129	Gamma	0
130	Gamma	0
131	Gamma	0

HPV type	Genus	Predicted C/EBPβ sites
132	Gamma	0
133	Gamma	0
134	Gamma	0
135	Gamma	0
136	Gamma	0
137	Gamma	1
138	Gamma	0
139	Gamma	0
140	Gamma	0
141	Gamma	0
143	Beta	1
144	Gamma	0
145	Beta	0
146	Gamma	0
147	Gamma	0
148	Gamma	0
149	Gamma	0
150	Beta	0
151	Beta	1
152	Beta	0
153	Gamma	0
154	Gamma	0
155	Gamma	0
156	Gamma	0
157	Gamma	0
158	Gamma	1
159	Beta	0
160	Alpha	1
161	Gamma	0
162	Gamma	0
163	Gamma	0
164	Gamma	0
165	Gamma	0
166	Gamma	0
167	Gamma	0
168	Gamma	0
169	Gamma	0
170	Gamma	0
171	Gamma	0
172	Gamma	0
173	Gamma	0
174	Beta	0
175	Gamma	0
178	Gamma	0
179	Gamma	0
180	Gamma	1
184	Gamma	0
187	Gamma	0
197	Gamma	0
199	Gamma	0
200	Gamma	0
201	Gamma	0
202	Gamma	1
203	Gamma	0
205	Gamma	0
209	Beta	1
213	Gamma	0
214	Gamma	0
215	Gamma	1
216	Gamma	0
223	Gamma	0
225	Gamma	0

Supplementary Table S2. List of the 186 HPV types whose LCRs were surveyed for the presence of putative C/EBPβ sites. The number and genus of each HPV type are indicated along with the number of C/EBPβ site(s) predicted in the LCR.

Supplementary References

1. Alvarez, J., Gagnon, D., Coutlee, F. & Archambault, J. Naturally Occurring Variations Modulate the Activity of the HPV33 Early Promoter and its Affinity for the E2 Transcription Factor. *Sci Rep* **8**, 15015, doi:10.1038/s41598-018-33243-y (2018).
2. Edgar, R. C. MUSCLE: multiple sequence alignment with high accuracy and high throughput. *Nucleic Acids Res* **32**, 1792-1797, doi:10.1093/nar/gkh340 (2004).
3. Waterhouse, A. M., Procter, J. B., Martin, D. M., Clamp, M. & Barton, G. J. Jalview Version 2 - a multiple sequence alignment editor and analysis workbench. *Bioinformatics (Oxford, England)* **25**, 1189-1191, doi:10.1093/bioinformatics/btp033 (2009).
4. O'Connor, M. J., Stunkel, W., Koh, C. H., Zimmermann, H. & Bernard, H. U. The differentiation-specific factor CDP/Cut represses transcription and replication of human papillomaviruses through a conserved silencing element. *J Virol* **74**, 401-410 (2000).
5. Bauknecht, T., Angel, P., Royer, H. D. & zur Hausen, H. Identification of a negative regulatory domain in the human papillomavirus type 18 promoter: interaction with the transcriptional repressor YY1. *The EMBO journal* **11**, 4607-4617 (1992).
